# Functional Characterization, Genome Assembly, and Annotation of *Geobacillus* sp. G4 Isolated from a Geothermal Field in Tacna, Peru

**DOI:** 10.3390/microorganisms13061374

**Published:** 2025-06-13

**Authors:** Alonso R. Poma Ticona, Karita C. R. Santos, Heber E. Ramirez-Arua, Roberto Castellanos, Jéssica Pinheiro Silva, Pedro R. Vieira Hamann, Eliane F. Noronha, Fabyano A. C. Lopes

**Affiliations:** 1 Enzyme Biotechnology Research Laboratory, Science Faculty, Universidad Nacional Jorge Basadre Grohmann, Tacna 23003, Peru; rcastellanosc@unjbg.edu.pe; 2 Laboratório de Microbiologia, Universidade Federal Do Tocantins, Porto Nacional 77500-000, TO, Brazil; karita.cristine@uft.edu.br; 3 Laboratorio de Biología Molecular, Facultad de Farmacia y Bioquímica, Universidad Nacional Mayor de San Marcos, Lima 15081, Peru; heber.ramirez@unmsm.edu.pe; 4 Department of Cell Biology, Institute of Biological Sciences, University of Brasilia, Brasilia 70910-900, DF, Brazil; wpinheiro.jessica@gmail.com (J.P.S.); pedrorvhamann@gmail.com (P.R.V.H.); enoronha@unb.br (E.F.N.)

**Keywords:** bacterial thermophily, biotechnological potential, industrial enzymes, phylogenomic analysis

## Abstract

The genome of *Geobacillus* sp. G4, a thermophilic bacterium isolated from a geothermal field in Peru, was sequenced and analyzed to evaluate its taxonomic and biotechnological potential. This strain exhibits optimal growth at temperatures between 50 and 70 °C and at a pH range of 6.0–7.5. Phenotypic assays demonstrated extracellular enzymatic activities, including amylases, cellulases, pectinases, and xylanases, highlighting its potential for efficient polysaccharide degradation. The assembled genome comprises approximately 3.4 Mb with a G+C content of 52.59%, containing 3,490 genes, including coding sequences, rRNAs, and tRNAs. Functional annotation revealed genes associated with key metabolic pathways such as glycogen and trehalose biosynthesis, indicating adaptation to carbohydrate-rich environments. Phylogenetic analyses based on ANI and dDDH values identified *Geobacillus thermoleovorans* KCTC3570 as its closest relative, suggesting a strong evolutionary relationship. Additionally, the genome harbors gene clusters for secondary metabolites such as betalactone and fengycin, suggesting potential industrial and pharmaceutical applications, including bioremediation. The identification of antibiotic resistance genes, specifically those conferring glycopeptide resistance, underscores their relevance for antimicrobial resistance studies. The presence of enzymes like amylases and pullulanase further emphasizes its biotechnological potential, particularly in starch hydrolysis and biofuel production. Overall, this research highlights the significant potential of *Geobacillus* species as valuable sources of thermostable enzymes and biosynthetic pathways for industrial applications.

## 1. Introduction

The genus *Geobacillus* comprises thermophilic, Gram-positive, spore-forming, rod-shaped bacteria that are either aerobic or facultatively anaerobic, constituting a phylogenetically coherent clade within the family Bacillaceae [1]. These microorganisms inhabit a wide range of environments, including hot springs, cold regions, and ocean sediments [2]. The genus *Geobacillus* was proposed by Nazina et al. (2001) [3] to differentiate it from the genus *Bacillus* based on distinct physiological traits, variations in growth temperatures, and phenotypic characteristics among strains. *Geobacillus* species exhibit broad catabolic versatility, facilitating their widespread occurrence across diverse environments. These bacteria are recognized for secreting commercially important enzymes, including lipases, xylanases, and amylases, widely utilized in various industrial and biotechnological processes.

According to the List of Prokaryotic Names with Standing in Nomenclature (LPSN, 2025), the genus *Geobacillus* currently includes 30 valid species, including *Geobacillus thermodenitrificans*, *Geobacillus uzenensis*, *Geobacillus thermocatenulatus*, *Geobacillus kaustophillus*, *Geobacillus thermoglucosidasius*, *Geobacillus thermoleovorans*, *Geobacillus subterraneus*, *Geobacillus stearothermophilus*, *Geobacillus toebii*, *Geobacillus jurassicus*, *Geobacillus thermoglucosidans*, *Geobacillus stearothermophilus*, and *Geobacillus thermodenitrificans. Geobacillus stearothermophilus* serves as the type species, with DSM 22 designated as its type strain [1,4].

Analysis based on the 16S rRNA gene revealed a cohesive grouping of 56 *Geobacillus* strains, exhibiting intra-species similarities between 99.3% and 100%, and inter-species similarities ranging from 96.0% to 99.4%, facilitating reliable distinctions [5]. However, limitations in taxonomic resolution using the 16S rRNA gene led researchers to adopt additional genetic markers, such as recN, infB, rpoB, and spo0A, providing enhanced phylogenetic clarity [6,7].

Distinctions between *Geobacillus* species in previous studies have been based on small chemotaxonomic differences and phenotypic characteristics [5]. Growth temperatures range from 35 °C to 80 °C, with most isolated strains growing between 45 °C and 70 °C [3]. The core genome of *Geobacillus* comprises approximately 1665 genes, 85% of which are shared with the genome of *Bacillus subtilis* 168, suggesting a common survival strategy centered around rapid growth and sporulation under nutrient-limited conditions [8].

Traditionally, bacterial classification has relied on methods such as 16S rRNA sequencing, DNA-DNA hybridization (DDH), and phenotypic characterization. Nonetheless, high sequence similarity within the 16S rRNA gene among *Geobacillus* species complicates precise identification and has resulted in misidentifications [9]. The growing number of studies classifying new species using only single-pathway markers has led to the rise of unreliable and confusing classifications within the genus [3,10,11,12]. To overcome these challenges, integrated approaches combining genomic and phenotypic analyses, including whole-genome sequencing, have been proposed to achieve higher phylogenetic resolution.

The Calientes geothermal field in Tacna, Peru, represents an underexplored source of microbial biodiversity with significant biotechnological potential [13,14]. Characterized by elevated temperatures and unique mineral compositions, these geothermal springs provide optimal conditions for thermophilic bacteria like *Geobacillus* [15,16]. These microorganisms have attracted considerable interest due to their ability to produce thermostable enzymes applicable in biotechnology, particularly in industrial processes involving the bioconversion and biodegradation of complex organic polymers such as lignocellulosic biomass (e.g., cellulose, hemicellulose, and lignin) [17].

*Geobacillus* species are highly valuable biotechnological resources due to their capability of producing stable and active enzymes under extreme environmental conditions, such as high temperatures and variable pH levels. These enzymes have found extensive industrial applications, notably in biofuel production and the bioconversion of industrial waste [18]. Moreover, certain *Geobacillus* strains efficiently degrade complex organic polymers like lignin and cellulose, offering promising opportunities for bioethanol production from renewable biomass [19]. Ongoing genomic and metabolic engineering research efforts further enhance the biotechnological potential of these bacteria, optimizing their utilization in industrial applications [20].

*Geobacillus* species have attracted considerable attention not only due to their capacity to produce thermostable enzymes but also because of their potential to biosynthesize secondary metabolites with diverse industrial and pharmaceutical applications. These metabolites include compounds like fengycin, known for its antimicrobial and antifungal properties, valuable in bioremediation and biological control applications, and lanthipeptides or betalactones, which exhibit potential therapeutic properties, including antibiotic and anticancer activities, relevant to pharmaceutical development [21]. The identification and characterization of secondary metabolite biosynthetic gene clusters (BGCs) in thermophilic bacteria can reveal novel compounds beneficial for biotechnological and biomedical use [17,18].

The study of genomic diversity and characterization of new *Geobacillus* strains is essential from both a taxonomic and biotechnological perspective. Genomic analysis of new strains allows for the identification of genes involved in specific metabolic processes, such as the degradation of starch and hemicellulose, which are crucial for biomass conversion into biofuels and other value-added products [22,23].

In this study, we aimed to characterize *Geobacillus* sp. G4, a thermophilic strain isolated from a geothermal environment in southern Peru, through phenotypic and enzymatic analyses. Furthermore, we sought to sequence, assemble, and annotate its genome; determine its taxonomic position; and identify genes and metabolic pathways with potential biotechnological relevance, particularly those involved in the production of thermostable enzymes and the degradation of complex carbohydrates for industrial applications.

## 2. Materials and Methods

### 2.1. Bacterium Isolation and Characterization of Geobacillus *sp.* G4

**Figure 1:**
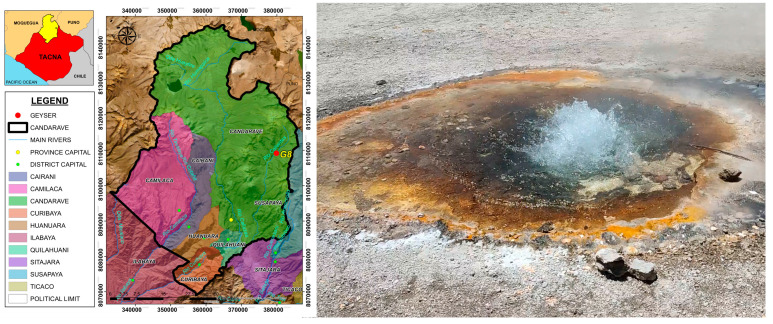
Map of the geothermal sampling area in Calientes, Candarave, using ArcGIS version 10.5. Image of the geothermal spring sampled in this study, located in the Calientes geothermal field, Candarave, Tacna, Peru.

Sediment samples were obtained from a geothermal spring in the Calientes geothermal field, Candarave, Tacna, Peru. The spring, designated as G8, was selected based on the presence of sediment, elevated temperatures (>60 °C), and accessibility. The G8 spring, with a water temperature of 65.3 °C and a pH of 7.35, is located at an altitude of 4162 m above sea level (UTM coordinates: 0.379908 E, 81.09185 N). The area surrounding the spring was characterized by sparse vegetation and the accumulation of brownish sediments (Figure 1). Approximately 250 g of sediment was collected from the upper 3 cm layer of the sediment bed, at a depth of up to 15 cm from the water surface, at the edge of the geothermal spring using a sterile plastic bottle. A suspension of sediment and spring water was transferred into a sterile thermos bottle to maintain a temperature close to that of the sample during both sampling and transport to preserve microbial viability.

A sediment sample (10 g) was initially enriched in Luria–Bertani (LB) liquid medium (consisting of 10 g/L peptone, 5 g/L yeast extract, and 10 g/L sodium chloride) at a 10% (*v*/*v*) concentration. The enrichment culture was incubated at 60 °C for 48 h. After incubation, 100 μL of the enriched culture (OD 600 nm = 0.2) was spread onto LB agar plates supplemented with 1% (*w*/*v*) starch as the sole carbon source to selectively isolate amylolytic bacteria. The plates were incubated at 60 °C until bacterial colonies appeared. After two days, bacterial colonies were individually collected and streaked three times on the same LB agar to ensure purity. Five amylolytic colonies (G1, G2, G4, G16, and G23) were isolated, and the strain G4, which exhibited the highest amylase activity levels, was chosen for further characterization and genomic analysis. Amylase activity was quantified using the Miller method [24]: 100 μL of starch solution (0.5% *w*/*v*) and 100 μL of G4 culture supernatant were incubated at 60 °C for 15 min. Then, 300 μL of DNS reagent was added, and absorbance was measured at 540 nm.

The morphology of the G4 strain was evaluated by analyzing colony shape, pigmentation, and microscopic characteristics using Gram staining, endospore formation assays, and motility tests. The effect of temperature on the growth of strain G4 was assessed by culturing it in LB liquid medium (pH 7) at a range of temperatures (37, 45, 50, 55, 60, 65, 70, 75, and 80 °C) for 48 h. Similarly, the effect of pH on growth was evaluated by incubating G4 in LB medium buffered at pH values ranging from 4 to 8 (100 mM sodium citrate for pH 4 and 5, 100 mM sodium phosphate for pH 6 and 7, and 100 mM Tris-HCl for pH 8) at a fixed temperature of 60 °C for 48 h. For acid production assays, substrates such as lactose, cellobiose, glycerol, sucrose, and xylose were used, following the methodology described by Mac Faddin (2000) [25].

Carbon utilization of strain G4 was determined by culturing it in LB liquid medium (pH 7) supplemented with various substrates, including carboxymethylcellulose (CMC), starch, pectin, microcrystalline cellulose, casein, lactose, xylose, glucose, sucrose, cellobiose, and maltose, each at 1% (*w*/*v*) as a carbon source (Sigma Aldrich, St. Louis, MO, USA). Cultures were incubated at 60 °C for 48 h. Bacterial growth was monitored by measuring optical density at 600 nm.

The extracellular hydrolytic enzyme activities, including cellulase, pectinase, xylanase, amylase, protease, and lipase, were assessed. The G4 isolate was cultivated on LB agar plates supplemented individually with specific substrates: carboxymethylcellulose (CMC) 1% (*w*/*v*), pectin 0.5% (*w*/*v*), oat spelt xylan 1% (*w*/*v*), potato starch 1% (*w*/*v*), casein 1% (*w*/*v*), and tributyrin 0.5% (*v*/*v*), following the protocol described by Guta et al. (2024) with minor modifications [26]. Plates were incubated at 60 °C for 24–48 h. The presence of clear zones surrounding colonies indicated positive enzymatic activity.

To analyze enzyme production by isolate G4, time-course experiments were performed. G4 was inoculated into 250 mL Erlenmeyer flasks containing 50 mL of LB liquid medium at pH 7.0, individually supplemented with the enzyme-specific substrates mentioned above. Cultures were incubated at 60 °C, and 2 mL samples were withdrawn at intervals of 3, 6, 12, 24, 30, and 48 h. Samples were centrifuged at 10,000× *g* for 10 min at 4 °C, and the resulting supernatants were used for enzyme assays.

The enzymatic activities of cellulase, pectinase, xylanase, and amylase were determined using their respective substrates buffered with 100 mM sodium phosphate (pH 7.0), quantifying the reducing sugars released according to the Ticona method [27]. One unit of enzymatic activity was defined as the amount of enzyme required to release 1 µmol of reducing sugar per minute.

Protease activity was assessed by mixing 25 µL of enzyme solution with 130 µL of 0.5% (*w*/*v*) casein buffered in 100 mM Tris-acetate (pH 7.5), following the methodology described by Oluoch et al. (2018) [28]. One unit of protease activity was defined as the amount of enzyme that released 1 µmol of tyrosine per minute. Lipase activity was measured titrimetrically using an olive oil emulsion as the substrate, as described by Sharma et al. (2017) [29]. One unit of lipase activity was defined as the enzyme amount releasing 1 μmol of fatty acid per minute under assay conditions.

### 2.2. DNA Extraction and Sequencing

Genomic DNA was extracted from bacterial strain G4 for whole-genome sequencing. Strain G4 was first cultured in 10 mL of Luria–Bertani (LB) broth and incubated at 60 °C for 48 h. The culture was centrifuged at 8000× *g* for 15 min at 4 °C, and the supernatant was discarded. The resulting cell pellet was resuspended in 5 mL of Tris-HCl buffer (pH 8.0). Genomic DNA was isolated using the Bacterial Genomic DNA Isolation Kit (Norgen Biotek Corp., Thorold, ON, Canada) following the manufacturer’s protocol. The concentration and purity of the DNA were measured using a BioTek Epoch 2T spectrophotometer (Agilent Technologies, Inc., Santa Clara, CA, USA).

DNA libraries were prepared using the Illumina TruSeq Nano DNA Library Prep Kit (350), and sequencing was performed using the Illumina NovaSeq platform with paired-end 150 bp reads at Macrogen Inc. (Seoul, Republic of Korea).

### 2.3. Quality Analysis, Genome Assembly, and Annotation of Geobacillus *sp.* G4

The raw fastq files (paired-end) comprising 13,778,078 sequences with an average length of 151 bp were imported into the FastQC v0.12.1 tool for quality assessment. The quality filtering was performed using Trimmomatic v0.38 to remove sequences with low-quality scores, applying the following parameters: SLIDINGWINDOW (4:30) and PHRED 30. The remaining sequences were then imported into the SPAdes v3.15.4 tool for genome assembly using the parameters k-mer (21, 33, 55, and 77) and a Phred score of 64 (Illumina). Short contigs (<200 bp) were removed from the assembly using seqtk v.1.4 (https://github.com/lh3/seqtk, accessed on 13 November 2024). The completeness and contamination of the genome were analyzed using the CheckM v1.2.2 tool and CoverM v0.7.0 (https://github.com/wwood/CoverM, accessed on 13 November 2024), while the size and quality of the assembled genome were assessed using Quast v5.2.0 and Busco v5.5.0 [30,31,32].

Functional genome annotation was performed in the Galaxy (https://usegalaxy.org/) environment using Prokka v1.14.6 with default parameters. Prokka predicted CDSs, rRNAs, and tRNAs, assigning functions initially through BLAST+ searches against core databases (ISfinder, NCBI AMR, and UniProtKB/SwissProt), followed by refinement using HMMER3 [33,34,35,36,37]. After annotation, the genome was mapped according to its functional categories and enzyme classes using the online mapper eggNOG-mapper v2 (eggNOG-mapper) with the eggNOG 5.0 database [38]. Type strains from the most similar species, based on the results from the Digital DNA-DNA hybridization (dDDH) analysis, were used for comparison with the literature.

To analyze and highlight the biotechnological potential of the *Geobacillus* sp. G4 strain, three metabolic pathways were highlighted in the KEGG annotation: glycogen biosynthesis (M00854), glycogen degradation (M00855), and trehalose biosynthesis (M00565) (Supplementary Figure S1). The visualization of the pathways of interest was performed using the KEGG mapper (www.genome.jp/kegg/mapper, accessed on 20 November 2024) via the KEGG database.

To identify gene clusters related to secondary metabolism (BCGs) in microorganisms, AntiSMASH v7.1.0 software was used [39]. The Antibiotic Resistance Genes Database (ARDB) was used to select and annotate genes potentially responsible for antibiotic resistance [40].

The genome of *Geobacillus* sp. G4, along with three reference genomes (*G. kaustophilus* HTA426, *G. kaustophilus* NBRC12445, and *G. thermoleovorans* KCTC3570), was functionally annotated using the eggNOG-mapper online tool (http://eggnog-mapper.embl.de/, accessed on 6 January 2025). Genes related to four functional categories of interest—carbon starvation, DNA repair and supercoiling, heat-shock response, and oxidative stress—were selected based on their presence in the genomes. Gene presence/absence analyses and graphical visualization were performed using R statistical software (https://www.r-project.org/).

### 2.4. Taxonomic Classification of Geobacillus *sp.* G4

#### 2.4.1. Digital DNA Hybridization (dDDH) and Average Nucleotide Identity (ANI)

Digital DNA hybridization (dDDH) was performed on the TYGS platform—DSMZ (tygs.dsmz.de) for the taxonomic classification of the strain and identity confirmation [41]. For phylogenomic inference to compare complete genomes in dDDH, the GBDP method (Genome BLAST Distance Phylogeny) was used, employing the trimming algorithm, formula d5, and bootstrap 100 [42]. To calculate genomic similarity using DDQ (Digital DNA-DNA Hybridization), GGDC v4.0 software integrated into the TYGS platform was used. The average nucleotide identity (ANI) analysis was calculated using the JSpeciesWS platform (https://jspecies.ribohost.com/), which provides ANIb, representing the average nucleotide identity based on BLAST calculations [43], and ANIm, representing the average nucleotide identity based on MUMmer calculations [43].

#### 2.4.2. 16S rRNA Gene-Based Phylogenetic Tree Reconstruction

16S rRNA gene sequences from the genomes were extracted using barnnap v0.9 (https://github.com/tseemann/barrnap, accessed on 11 January 2025). All information regarding the sequences used can be found in Supplementary Table S1. Sequence alignment was performed using MAFFT v7.526 in default mode [44]. After alignment, all sequences were trimmed using TrimAl v1.4.1 [45]. The selection of the most appropriate model for tree reconstruction was performed using ModelTest-NG v0.1.7 [46], suggesting the TIM1+I model. The phylogenetic tree was constructed using the Maximum Likelihood method [47], utilizing RAxML-NG X v1.2.2 [48] with the following parameters: 10 random initial parsimony trees, nucleotide substitution matrix model (TIM1+I), and 1000 bootstrap replications. The *Bacillus subtilis* DSM10 strain was used as an outgroup.

#### 2.4.3. Phylogenetic Tree Reconstruction

The genomes used were obtained from complete genomes deposited in GenBank. The selection of type strains for valid *Geobacillus* and *Bacillus* species used was based on the LPSN (List of Prokaryotic names with Standing in Nomenclature—lpsn.dsmz.de) [41,49]. All information regarding the complete genomes used can be found in Supplementary Table S2.

Two phylogenetic trees were constructed using the studied genome. The first tree was reconstructed using molecular markers extracted from the genomes using AMPHORA2 [50], and data on the extracted markers are available in Supplementary Table S3. Sequence alignment was performed using the MAFFT v7.526 aligner in default mode [44]. After alignment, all sequences were trimmed using TrimAl v1.4.1 [45]. The selection of the most appropriate model for tree reconstruction was performed using ModelTest-NG v0.1 [46], with the LG+I+G4+F model suggested. The phylogenetic tree was constructed using the Maximum Likelihood method [47], utilizing RAxML-NG X v1.2.2 [48] with the following parameters: 10 random initial parsimony trees, nucleotide substitution matrix model (LG+I+G4+F), and 1000 bootstrap replications. The *Bacillus subtilis* DSM10 strain was used as an outgroup.

The second tree was reconstructed using the FastME v2.1.6.1 tool [51] with the GBDP method (Genome BLAST Distance Phylogeny) integrated into the TYGS platform (tygs.dsmz.de). The clade lengths are scaled in terms of GBDP distance formula d5, and 100 pseudo-bootstrap replicates were performed. Only pseudo-bootstrap support values above 60% were accepted. The tree was rooted at the midpoint [52]. The *Bacillus subtilis* DSM10 strain was used as an outgroup.

For genome comparison, the online platform Proksee (proksee.ca) was used [53]. Comparisons were made using the genome of the type strains *G. thermoleovorans* KCTC3570 and *G. kaustophilus* NBRC102445 in comparison with the *Geobacillus* sp. G4 strain. BLAST v1.3.1 and BLAST Formatter v1.0.3 tools were used to check identity and similarity profiles between the genomes [42].

## 3. Results

### 3.1. Characterization of Geobacillus *sp.* G4

The *Geobacillus* sp. G4 strain is a Gram-positive, rod-shaped bacterium forming round, white-yellow colonies with an average diameter of 1.4 mm (Supplementary Table S4). Characterization revealed that *Geobacillus* sp. G4 is aerobic, non-motile, and forms endospores. Optimal growth conditions (50 °C to 70 °C and pH 6.0 to 7.5) were determined in LB liquid medium based on maximal bacterial growth, as measured by optical density at 600 nm. Additionally, acid production from lactose, cellobiose, glycerol, sucrose, and xylose was observed (Table 1).

**Figure 2:**
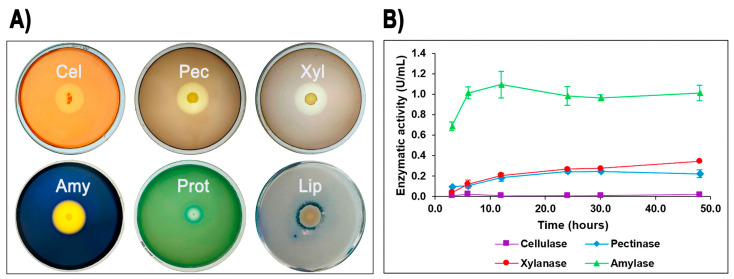
Quantitative and qualitative enzymatic activity of *Geobacillus* sp. G4. Extracellular enzymatic activities of *Geobacillus* sp. G4. (**A**) Formation of clear halos on agar plates indicating positive extracellular enzyme production: Amy (amylase), Pec (pectinase), Xyl (xylanase), Cel (cellulase), Prot (protease), and Lip (lipase). (**B**) Enzyme production profiles measured in LB liquid media over time.

The ability of isolate G4 to produce extracellular hydrolytic enzymes was evaluated on agar plates. After incubation at 60 °C for 24–48 h, isolate G4 demonstrated positive enzymatic activities for cellulase, pectinase, xylanase, amylase, protease, and lipase, indicated by distinct clear zones around colonies. Among these, *Geobacillus* sp. G4 showed the largest halo formation for amylase (starch), followed by pectinase, xylanase, and cellulase (Figure 2A).

To further quantify these enzymatic activities, isolate G4 was cultured in liquid media containing potato starch, oat spelt xylan, pectin, carboxymethylcellulose (CMC), olive oil, and casein as carbon sources. Enzyme assays revealed measurable production of amylase (maximum activity: 1.09 U/mL at 12 h), xylanase (0.34 U/mL at 48 h), and pectinase (0.24 U/mL at 30 h), as well as low but detectable cellulase activity (0.02 U/mL at 48 h) (Figure 2B). In contrast, protease and lipase activities, although clearly evident in agar plate assays, were below detection limits in liquid media under the tested conditions.

### 3.2. Genome Description and Taxonomic Classification of Geobacillus *sp.* G4

The results regarding the preliminary genome draft of *Geobacillus* sp. G4 is presented in Table 2. The strain genome has a size of approximately 3.4 Mb with a coverage of ~1225x. The assembly comprised 164 contigs, with a total G+C content of 52.59%. The genome completeness and contamination were 99.45% and 0.95%, respectively. The N50 and L50 values for the assembly were 236,119 bp and 5 contigs, respectively. The annotation identified a total of 3490 genes: 2047 coding sequences (CDS), 5 rRNAs, 88 tRNAs, 1 transfer-messenger RNA, and 1349 putative proteins. Regarding the gene clusters related to secondary metabolism (BCGs), seven clusters were identified: betalactone, T3PKS, Lanthipeptide class-i, NI-siderophore, terpene, fengycin, and RiPP-like.

The nucleotide average identity (ANI) values between the *Geobacillus* sp. G4 genome and other species of the *Geobacillus* genus show similarity with *Geobacillus kaustophilus* and *Geobacillus thermoleovorans*, with ANI values above 97% (Table 3). Thus, the obtained results suggest that *Geobacillus thermoleovorans* KCTC3570 and *Geobacillus* sp. G4 are sufficiently similar to be considered as belonging to the same species.

In the dDDH analysis, the *Geobacillus* sp. G4 strain showed dDDH values above 70% with *Geobacillus kaustophilus* HTA426 (79.1%) and *Geobacillus kaustophilus* NBRC102445 (78.5%) (Table 4). However, the *Geobacillus thermoleovorans* KCTC 3570 strain exhibited the highest similarity with *Geobacillus* sp. G4, with a dDDH value of 83.2% and a GC content difference of only 0.31%, shows a high genomic proximity. This suggests that both organisms are closely related, meaning *Geobacillus* sp. G4 could be a variant of *G. thermoleovorans*. Other species showed dDDH values below 70% and a larger GC content difference. These data indicate that, among the species compared, *Geobacillus thermoleovorans* is the closest to *Geobacillus* sp. G4 in terms of genomic similarity.

The 16S rRNA gene sequence of the *Geobacillus* sp. G4 strain showed 99.87% identity with *Geobacillus kaustophilus* strain BGSC 90A1 and 99.80% identity with *Geobacillus thermoleovorans* strain LEH-1 (Supplementary Table S5). The *Geobacillus thermoleovorans* BGSC 96A1 lineage also had 100% query coverage and 99.61% identity. Other *Geobacillus* and *Bacillus* strains showed identity percentages ranging from 99.08% to 99.87%, with query coverages ranging from 92% to 100%.

**Figure 3:**
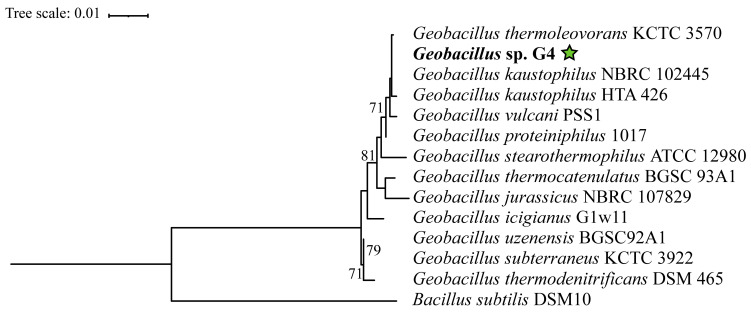
Phylogenetic tree based on the 16S rRNA gene of 13 type strains of *Geobacillus* and *Bacillus*, extracted from the complete genome using the (barrnap) tool. Accession numbers and references are available in Supplementary Table S1. Evolutionary analyses were conducted using the RAxML tool. The strain *Geobacillus* sp. G4, in bold, corresponds to the 16S gene from the genome assembled in this study. The green star indicates the position of the strain isolated in this study.

In the phylogenetic tree constructed based on the 16S rRNA gene, the *Geobacillus* strains are distributed across several clades without statistical support (Figure 3), indicating significant diversification within this genus. However, *Geobacillus* sp. G4 is positioned within a clade supported by a bootstrap value of 71%, containing the strains *Geobacillus thermoleovorans* KCTC3570, *Geobacillus kaustophilus* HTA426, *Geobacillus kaustophilus* NBRC102445, *Geobacillus proteiniphilus* 1017, and *Geobacillus vulcani* PSS1.

**Figure 4:**
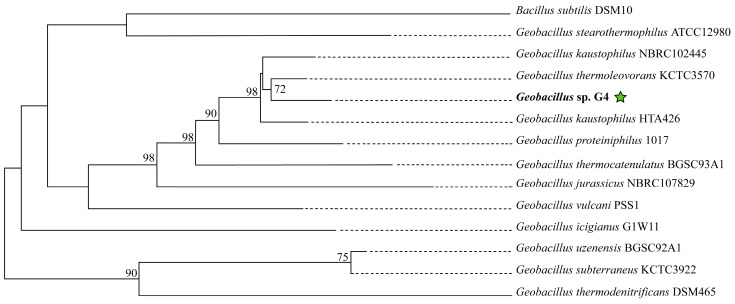
Phylogenetic tree of 13 type strains of *Geobacillus* and *Bacillus*, constructed on the online platform TYGS available at (https://tygs.dsmz.de). The green star indicates the position of the strain isolated in this study.

In the phylogenomic tree reconstructed using molecular markers extracted with AMPHORA2, the strain *Geobacillus* sp. G4 is grouped with *Geobacillus kaustophilus* HTA426 and *Geobacillus thermoleovorans* KCTC3570, forming a clade supported by a bootstrap value of 95% (Supplementary Figure S1). In the phylogenomic tree reconstructed on the TYGS platform (Figure 4), the strain *Geobacillus* sp. G4 is in the clade with the strain *Geobacillus thermoleovorans* KCTC3570, supported by a bootstrap of 72%.

**Figure 5:**
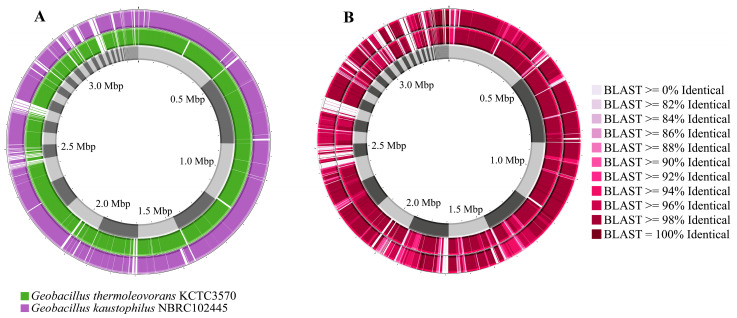
Comparison of the genomes of *G. thermoleovorans* KCTC3570 and *G. kaustophilus* NBRC102445 with the genome of the isolated strain *Geobacillus* sp. G4. (**A**) *Geobacillus* sp. G4 genome (gray), *G. thermoleovorans* KCTC3570 genome (green), and *G. kaustophilus* NBRC102445 genome (purple). (**B**) Similarity comparison between the genomes of *Geobacillus* sp. G4 (gray), *G. thermoleovorans* KCTC3570 (inner circle), and *G. kaustophilus* NBRC102445 (outer circle).

Comparing the genomes of *Geobacillus* sp. G4 with the genomes of *G. kaustophilus* NBRC102445 and *G. thermoleovorans* KCTC3570 reveals several gaps between them, with a higher frequency observed against *G. kaustophilus* NBRC102445 (Figure 5A). It was also noted that *Geobacillus* sp. G4 showed greater similarity across several regions of its genome compared to *G. thermoleovorans* KCTC3570 (Figure 5B).

### 3.3. Functional Analysis of Geobacillus thermoleovorans G4 Strain

**Figure 6:**
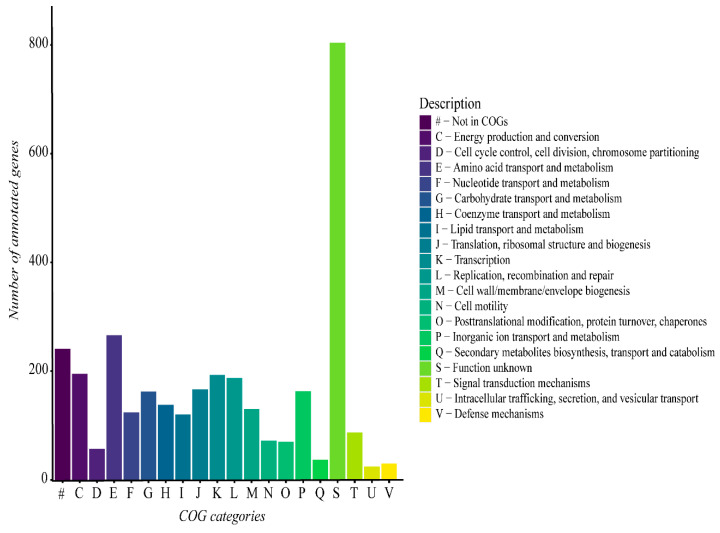
Functional gene categories derived from the functional annotation of the *G. thermoleovorans* G4 genome using Prokka software and the UniProt database on the Galaxy server. Categories were classified using the eggNOG-mapper online tool with the eggNOG5 database.

The functional analysis of the *G. thermoleovorans* G4 genome revealed a predominance of genes involved in different various metabolic categories, including metabolism, transport, transcription, replication, and repair, among others (Figure 6). Additionally, a large portion of genes were not classified into any known COG category, highlighting the presence of genes with functions that remain unclear within the context of the *G. thermoleovorans* G4 genome.

**Figure 7:**
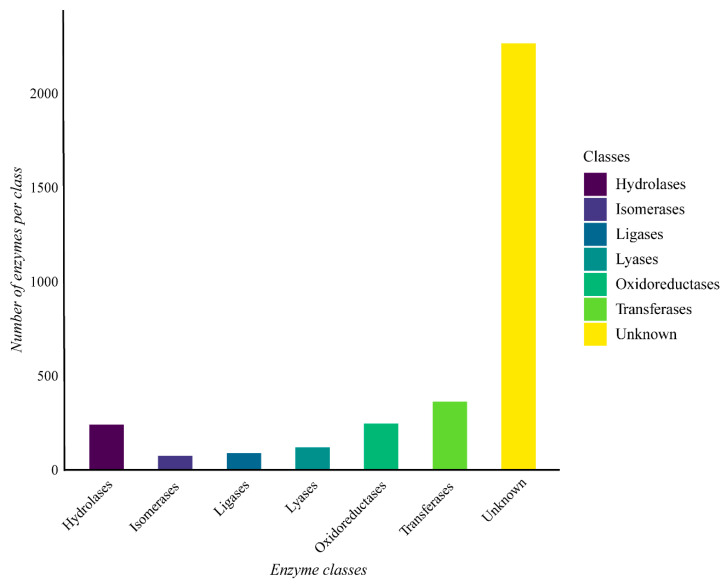
Enzyme classes were obtained through functional annotation of the assembled genome of *Geobacillus thermoleovorans* G4 using Prokka software on the Galaxy server (https://usegalaxy.org/) and classified using the eggNOG-mapper online tool with the eggNOG 5 database (http://eggnog-mapper.embl.de/). The “Unknown” category refers to proteins encoded in the genome that have been annotated as possible enzymes, but do not have an assigned EC number.

The genome of *G. thermoleovorans* G4 shows a predominance of enzymes belonging to the various classes, with hydrolases, transferases, and oxidoreductases being the most abundant (Figure 7). Additionally, many sequences lacked known annotations for any of the classes.

**Figure 8:**
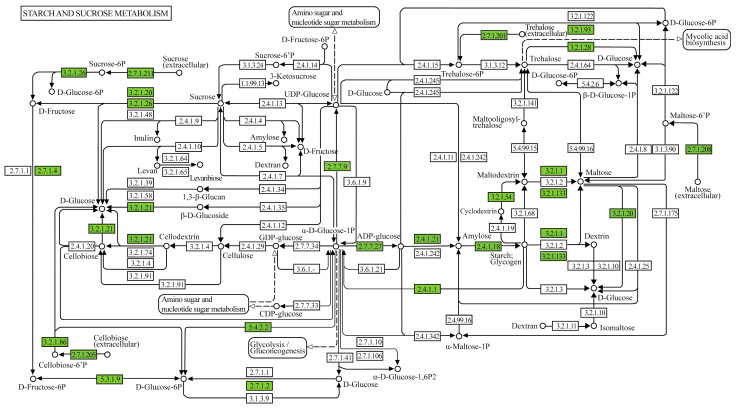
Metabolic pathway map of starch and sucrose metabolism displaying genes and enzymes of interest annotated in the genome of *G. thermoleovorans* G4. Annotations were performed using the KEGG Mapper tool (www.genome.jp/kegg/mapper, accessed on 20 November 2024), identifying key enzymes involved in the metabolism of sugars and complex carbohydrates.

In the set of metabolic pathways involved in the degradation of starch and sucrose present in the genome of *Geobacillus thermoleovorans* G4, enzymes involved in the degradation, conversion, and biosynthesis of sugars such as glucose, fructose, and their phosphorylated derivatives were observed (Figure 8). In the glycogen biosynthesis pathway (Supplementary Figure S2A), the enzymes annotated were 1,4-alpha-glucan branching enzyme (GBE1, glgB), glucose-1-phosphate adenylyltransferase (glgC), UTP-glucose-1-phosphate uridylyltransferase (UGP2, galU, galF), and starch synthase (glgA), all classified under transferases, related to starch synthesis. In the glycogen degradation pathway (Supplementary Figure S2B), the annotated enzymes were glycogen phosphorylase (PYG, glgP), pullulanase (pulA), phosphoglucomutase (pgm), and phosphomannomutase/phosphoglucomutase (pmm-pgm), classified under transferases, isomerases, and hydrolases, related to the mobilization and breakdown of glycogen and starch. In the trehalose biosynthesis pathway (Supplementary Figure S2C), the annotated enzymes were glucose-1-phosphate adenylyltransferase (glgC), starch synthase (glgC), and 1,4-alpha-glucan branching enzyme (GBE1, glgB), classified under transferases, related to starch synthesis.

The analysis of antibiotic resistance genes in the genome of *G. thermoleovorans* G4 identified two clusters related to glycopeptide resistance: vanY/vanB_cl and vanT/vanG_cl (Supplementary Table S6). The similarity of these genes between *Geobacillus* sp. G4 and other species ranged from 34.15% to 36.41% for vanY/vanB_cl and from 35.9% to 34.15% for vanT/vanG_cl, with both species compared showing high similarity.

For the *G. thermoleovorans* G4 strain, clusters for seven different types of secondary metabolites were identified: betalactone, T3PKS, Lanthipeptide class-i, NI-siderophore, terpene, fengycin, and RiPP-like (Supplementary Table S7). Comparatively, the strains *Geobacillus kaustophilus* HTA425 (G.K HTA425), *Geobacillus kaustophilus* NBRC102445 (G.K NBRC102445), and *Geobacillus thermoleovorans* KCTC3570 (G.T KCTC3570) also presented clusters for some of the analyzed secondary metabolites, except for NI-siderophore, fengycin, and RiPP-like, where no clusters were found. These observations suggest a shared biosynthetic capability among the strains, with minimal variations in the types of secondary metabolites detected.

**Figure 9:**
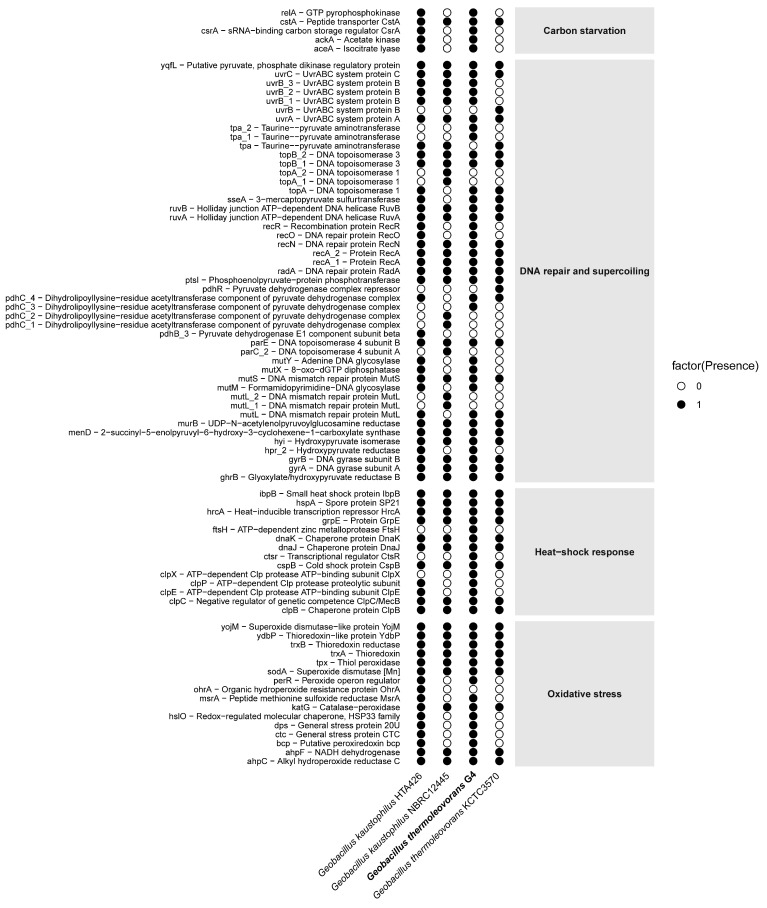
Distribution of presence/absence of stress-related genes in the genome of *G. thermoleovorans* G4 and in reference genomes of other *Geobacillus* strains. Each row represents an annotated gene, while each column corresponds to a strain. Black circles indicate the presence of the gene, and white circles indicate its absence. Genes are grouped into four main functional categories: carbon starvation, DNA repair and supercoiling, heat-shock response, and oxidative stress.

The *Geobacillus thermoleovorans* G4 strain presents a complete repertoire of genes (Figure 9) associated with carbon starvation, including *relA*, *cstA*, *ackA*, and *accA*. Complementarily, in the DNA repair and supercoiling category, a diverse set of genes was observed, such as *uvrABC*, *recA*, *recO*, *recR*, *mutS*, *mutL*, and *gyrAB*. In addition, classical heat-shock response genes were detected, including *hrcA*, *dnaK*, *groEL*, *clpB*, and *ibpB*. Finally, the presence of oxidative stress-related genes, such as *sodA*, *katA*, *ahpC*, *trxB*, and *msrA*, was also confirmed. The complete list of selected genes, along with their corresponding COG reference numbers, can be found in Supplementary Table S8.

## 4. Discussion

The genomic characterization and functional analysis of *Geobacillus* sp. G4, isolated from the geothermal field of Calientes in Tacna, Peru, provided valuable insights into its biotechnological potential. *Geobacillus* sp. G4 exhibited morphological and physiological characteristics typical of the *Geobacillus* genus. Initially, a direct comparison with *Geobacillus kaustophilus* and *Geobacillus thermoleovorans* was established, given that preliminary phylogenetic analyses and previous studies identified these species as the closest phylogenetic relatives to strain G4, sharing similar physiological profiles and temperature ranges [1,46].

Morphologically, strain G4 is characterized as a Gram-positive, endospore-forming rod, producing yellowish-white colonies approximately 1.4 mm in diameter, features that are commonly observed among thermophilic *Geobacillus* strains [3,5]. Its growth within a temperature range of 50–70 °C and an optimal pH of 6.0–7.5 further confirms its adaptation to thermophilic environments.

Table 1 specifically compares the properties of G4 with *G. kaustophilus* and *G. thermoleovorans*, highlighting relevant similarities and differences. All three strains are aerobic, Gram-positive, non-motile, and endospore-forming; however, notable differences were observed regarding acid production from various substrates. For instance, *G. kaustophilus* does not produce acid from lactose, whereas G4 does, along with cellobiose, glycerol, sucrose, and xylose, indicating broader or more efficient metabolic pathways for sugar degradation. The shared ability of G4 and *G. thermoleovorans* to metabolize lactose and glycerol supports their close relationship [1].

Regarding the optimal pH range, *Geobacillus* sp. G4 exhibits growth between pH 6.0 and 7.5, slightly narrower compared to some *G. thermoleovorans* strains that can tolerate conditions up to approximately pH 9.0. Additionally, the tolerance of strain G4 to NaCl concentrations up to 2.5% (*w*/*v*) indicates adaptations to moderately saline environments, a trait shared by many thermophilic *Geobacillus* species inhabiting geothermal springs [19,54,55].

*Geobacillus* sp. G4 exhibited extracellular enzymatic activities for cellulase, pectinase, xylanase, amylase, protease, and lipase on agar plates, with notably prominent hydrolysis halos observed particularly on starch-containing media. Similar enzyme profiles were previously reported for other thermophilic strains, such as *G. stearothermophilus*, *Geobacillus* sp. GS33, *Geobacillus* sp. WSUCF1, and *Geobacillus* sp. DUSELR13, particularly regarding the stability of amylase and xylanase at elevated temperatures [56,57,58,59]. Although protease and lipase activities were detectable in solid media assays, they were not quantifiable in liquid cultures, potentially due to differences in gene regulation or specific culture conditions [60,61].

Moreover, the diverse enzymatic profile identified in *Geobacillus* sp. G4 highlights a promising opportunity for developing enzyme cocktails tailored for efficient lignocellulosic biomass conversion into bioethanol and other valuable bioproducts. Despite their recognized biotechnological potential, relatively few studies have focused on formulating enzyme cocktails based on multiple thermostable enzymes derived from *Geobacillus* species [2,18].

Genome sequencing revealed a 3.4 Mb genome with a G+C content of 52.59%, consistent with genomic attributes previously reported for the *Geobacillus* genus, including strains such as *Geobacillus stearothermophilus* EF60045 and SJEF4-2 and *Geobacillus* sp. E55-1, and *Geobacillus* sp. 56T2 and 56T3 [22,62,63]. Studies demonstrate that the pan-genome of these bacteria reveals great genetic diversity, facilitated by horizontal gene transfer (HGT), which enables adaptation to various ecological niches [64]. However, classification based solely on 16S rRNA sequences has proven to be limited due to the high similarity between species [9]. To address this issue, ANI and dDDH analyses, combined with complete genome sequences, provide more precise phylogenetic resolution [65].

In our study, ANI and dDDH analyses revealed that *Geobacillus* sp. G4 is closely related to *G. thermoleovorans* KCTC3570, with ANI values above 97% and dDDH values exceeding 83%. These results are consistent with previous studies indicating that species such as *G. kaustophilus* and *G. thermoleovorans* could be conspecific, suggesting ongoing taxonomic revisions [66]. Phylogenetic analysis corroborates this relationship, showing that *Geobacillus* sp. G4 aligns within the *G. thermoleovorans* clade, indicating an evolutionary proximity.

In the genome of *G. thermoleovorans* G4, key enzymes for the degradation of complex carbohydrates, such as amylases and pullulanase, were identified. These enzymes play essential roles in breaking down starch and pullulan into simpler sugars. These enzymes are widely reported in other *Geobacillus* strains [57], reflecting the metabolic versatility of this bacterium in starch and sucrose degradation.

The enzymatic classes of hydrolases, transferases, and oxidoreductases are essential for the metabolism and adaptation of *Geobacillus* to extreme environments. Hydrolases enable the breakdown of biomolecules and the acquisition of nutrients under limited conditions. Transferases regulate biosynthesis and metabolic pathways, facilitating environmental adaptation. Oxidoreductases are crucial for energy production and defense against oxidative stress. The abundance of these enzymes in the *G. thermoleovorans* sp. G4 genome highlights its ability to adapt and survive in harsh environments.

Concerning the biotechnological potential of *G. thermoleovorans*, extensive research has focused on identifying enzymes capable of withstanding extreme environmental conditions, such as elevated temperatures and the presence of inhibitory substances [67,68]. Several enzymes from extremophiles have been identified as interesting tools for industrial processes, including biomass deconstruction in biorefinery [69,70].

Here, we describe a new strain of *G. thermoleovorans* with the potential to produce several industrially relevant enzymes, including more specific endoglucanases (evaluated against carboxymethylcellulose) and pectinases. Cellulases derived from organisms capable of surviving extreme temperatures are extensively documented targets for biotechnological applications because of their ability to withstand prolonged exposure to high temperatures while maintaining catalytic activity [71,72]. The capacity of *G. thermoleovorans* to grow in the presence of pectin highlights its potential to degrade other complex carbohydrates present in plant cell walls.

Despite an increasing number of reports describing thermophiles that can grow in the presence of cellulose-based polymers, there are few studies describing such organisms as capable of growing in the presence of other complex carbohydrates, such as pectin. The ability of *G. thermoleovorans* to grow in the presence of pectin may indicate its diverse potential to degrade such complex polymers, which require enzymes with different modes of action, including esterases, lyases, and glycosyl hydrolases [73,74]. To date, the potential of this bacterium to degrade complex carbohydrates has not been explored; however, closely related species have been described as potential producers of pectin lyases [75].

The strain explored in the present study could grow in the presence of starch, which may be a direct consequence of the diversity of starch-degrading enzymes produced by this organism. *G. thermoleovorans* has been reported to be a good producer of hyperthermostable amylases, including maltogenic α-amylase [76]. The results shown in the present study and previous literature call attention to further heterologous expression and characterization of such enzymes with untapped biotechnological potential.

Genes involved in metabolic categories such as nutrient metabolism, molecule transport, transcription, DNA replication, and repair are fundamental to ensuring the survival and adaptation of *Geobacillus* sp. G4 in extreme environments [18]. Nutrient metabolism and molecule transport ensure that the bacterium can absorb and process a wide variety of carbon and energy sources, which is essential in habitats with limited resources or adverse conditions. Genes related to transcription and replication ensure the regulation and efficient reproduction of genetic material, while DNA repair genes are crucial for maintaining genomic integrity, especially in environments that expose the organism to stress factors such as heat, radiation, or oxidative compounds [77].

The presence of pathways for glucose-6-phosphate and fructose-6-phosphate metabolism highlights the adaptation of *G. thermoleovorans* G4 to carbohydrate-rich environments. Furthermore, enzymes associated with cellulose and cellobiose processing indicate a robust capacity to degrade lignocellulosic materials, an important resource for biofuel production, as well as efficient adaptation for energy production via glycolysis, reflecting the metabolic versatility and adaptation of *Geobacillus* to various ecological niches, including extreme environments [78].

The *G. thermoleovorans* G4 strain also harbors genes related to antibiotic resistance, such as the vanY/vanB_cl and vanT/vanG_cl clusters, associated with glycopeptide resistance [79], with similarity ranging from 34% to 36% with other *Geobacillus* species. These findings suggest potential for studies on antimicrobial resistance, which could have important implications for understanding the evolution of bacterial resistance.

This study highlights the identification of seven biosynthetic gene clusters associated with secondary metabolites, including betalactones, terpenes, and, notably, fengycin. Fengycin is a well-known antimicrobial lipopeptide typically produced by mesophilic *Bacillus* species; however, its occurrence in thermophilic *Geobacillus* species has not been previously reported [80,81]. Studying fengycin from *G. thermoleovorans* G4 could uncover novel structural features, enhanced stability, or unique biological activities, highlighting its potential for innovative industrial and pharmaceutical applications.

During carbon starvation, bacteria activate specific mechanisms, including the expression of genes such as *cstA*, *relA*, and *spoT*, to maintain cellular viability. The *cstA* gene encodes a peptide transporter that is upregulated during nutrient limitation and regulated by *CsrA*, while *relA* and *spoT* mediate the stringent response through the synthesis of (p)ppGpp, which modulates global gene expression and protein synthesis to adapt to stress conditions [82,83,84].

To preserve genomic integrity, *G. thermoleovorans* G4 possesses conserved DNA repair systems such as nucleotide excision repair (NER) and mismatch repair (MMR). NER, involving *uvrA*, *uvrB*, and *uvrC*, is critical for correcting UV-induced lesions and bulky adducts [85]. Likewise, MMR, mediated by *mutS* and *mutL*, ensures replication fidelity by removing base mismatches [86]. Extremophilic organisms have adapted these systems with functional versatility to cope with high-stress environments.

Oxidative stress responses in *G. thermoleovorans* G4 include enzymes such as superoxide dismutase (*sodA*), catalase (*katA*), and alkyl hydroperoxide reductase (*ahpC*), which detoxify reactive oxygen species (ROS). The overexpression of *ahpC*, for instance, enhances tolerance to oxidative damage, as shown in other bacterial models [87].

Under heat stress, genes such as *dnaK*, *groEL*, and *clpXP* are upregulated, functioning as molecular chaperones and proteases that maintain protein folding and quality control. These genes are regulated by the transcriptional repressor *hrcA*, which functions as a protein thermosensor in thermophilic organisms [88].

Finally, these genomic adaptations spanning nutrient limitation, DNA repair, and oxidative and thermal stress responses emphasize the ecological resilience of *G. thermoleovorans* G4 and its promising value for industrial applications requiring stability under extreme environmental conditions.

## 5. Conclusions

The genomic and functional data of *Geobacillus thermoleovorans* G4 demonstrate its value as a highly promising strain for biotechnological applications. Its thermostable enzymes, along with biosynthetic diversity and antibiotic resistance genes, make this strain an important target for biofuel production, industrial biotechnology, and antimicrobial resistance studies.

The detailed analysis of the *G. thermoleovorans* G4 genome reinforces the importance of combining modern molecular tools, such as ANI, dDDH, and phylogenomics, for accurate bacterial taxonomy and understanding of phylogenetic relationships within the *Geobacillus* genus. This not only improves the classification of new strains but also facilitates the development of innovative biotechnological solutions based on the adaptive and metabolic capabilities of these extremophilic microorganisms.

## Figures and Tables

**Table 1:** Phenotypic characteristics for differentiation among *G. kaustophilus*, *G. thermoleovorans*, and *Geobacillus* sp. G4.

Phenotypic Characteristics	*Geobacillus* sp. G4 *	*G. kaustophilus* ATCC 8005 **	*G. thermoleovorans* ATCC 43513 **
Gram Staining	Positive	Positive	Positive
Motility	Non-motile	Non-motile	Non-motile
Morphology	Rod shape	Rod shape	Rod shape
Oxygen Requirement	Aerobic	Aerobic	Aerobic
G+C (%)	52	51–58	52–58
Endospore Formation	+	+	+
Voges-Proskauer	-	-	-
Methyl red	+	+	no data
Catalase	+	+	+
Oxidase	+	+	+
Hydrolysis of:			
Casein	+	+	Variable
Starch	+	+	+
Acid from:			
Lactose	+	-	+
Cellobiose	+	+	Variable
Glycerol	+	+	+
Sucrose	+	+	Variable
Xylose	+	+	Variable
Growth at/in:			
pH range	6.0–7.5	6.2–7.5	5.0–9.0
NaCl (%, *w*/*v*)	0–2.5	0–2	0–4
Temperature range (°C)	50–70	40–75	37–70
Acetate	+	+	+
Citrate	-	+	-

* Unpublished data; ** from Najar and Thakur (2020) [1].

**Table 2:** Genome assembly data for *Geobacillus* sp. G4 was performed using SPAdes v.3.15.4. The annotation was conducted using Prokka v.1.14.6, and quality parameters were assessed using Busco v.5.5.0 and Quast v.5.2.0. All processes were carried out on the Galaxy v24.1.2 server.

General Data	Assembly De Novo *Geobacillus* sp. G4	Reference Genome *G. kaustophilus* HTA426	Reference Genome *G. thermoleovorans* KCTC 3570	Reference Genome *G. kaustophilus* NBRC102445
*Contigs*	164	2	2	1
Total length	3,362,411	3,544,776	3,450,609	3,670,957
N50 (KB)	236,119	3,716,831	1,192,538	750,728
L50	5	1	2	1
GC (%)	52.59	52.35	52.50	51.73
Coverage	1224.95	-	224.2	150.5
Completeness (%)	99.45	99.31	99.44	93.81
Contamination (%)	0.95	0.22	30.89	0.24
CDS	2047	5668	4714	5548
rRNA	5	37	32	35
tRNA	88	129	100	111
tmRNA	1	2	1	3
Genes	2047	3655	3622	3734
Putative protein	1349	3386	3369	3401

**Table 3:** Average nucleotide identity (ANI) calculated for *Geobacillus* sp. G4 using type strains of each species of the *Geobacillus* genus, calculated on the JSpeciesWS platform (ANIb and ANIm). Information is available at the following link: (https://jspecies.ribohost.com/jspeciesws/, accessed on 11 January 2025).

Isolated Strain	Type Reference Strains	ANIb (%)	ANIm (%)
	*G. thermoleovorans* KCTC3570	97.65	98.30
	*G. kaustophilus* HTA426	97.19	97.91
	*G. kaustophilus* NBRC102445	97.11	97.85
*Geobacillus* sp. G4	*G. proteiniphilus* 1017 *G. thermocatenulatus* BGSC93A1 *G. jurassicus* NBRC107829 *Geobacillus vulcani* PSS1	94.66 93.15 90.57 90.41	95.51 94.06 91.52 91.54
	*G. stearothermophilus* ATCC12980 *G. uzenensis* BGSC92A1	88.93 85.05	90.65 87.16
	*G. subterraneus* KCTC3922 *G. icigianus* G1w11	85.04 84.64	87.12 87.28
	*G. thermodenitrificans* DSM465	82.64	85.04

**Table 4:** Digital DNA hybridization (dDDH) generated by the TYGS-DSMZ platform between the *Geobacillus* sp. G4 genome and the species described for the genus up to August 2024. All type strains of each species according to the List of Prokaryotic Names with Standing in Nomenclature (https://lpsn.dsmz.de/, accessed on 9 January 2025).

*Geobacillus* sp. G4	*dDDH Formula 4-d_4_ (Similarity Based on Sequence Identity)*		
Reference Genomes	dDDH (d_4_%)	Model C.I. (%)	G+C Difference (%)
*G. thermoleovorans* KCTC3570 *G. kaustophilus* HTA426	83.2 79.1	[80.4–85.7] [76.2–81.8]	0.31 0.61
*G. kaustophilus* NBRC102445	78.5	[75.6–81.2]	0.74
*G. proteiniphilus* 1017 *G. thermocatenulatus* BGSC93A1	61.5 53.4	[58.6–64.3] [50.7–56.1]	0.84 0.82
*G. vulcani* PSS1	43.3	[40.8–45.9]	0.19
*G. jurassicus* NBRC107829	42.8	[40.3–45.4]	0.38
*G. stearothermophilus* ATCC12980	38.7	[36.2–41.2]	0.18
*G. uzenensis* BGSC92A1	30.4	[28.1–32.9]	0.37
*G. subterraneus* KCTC3922	30.6	[28.2–33.1]	0.4
*G. icigianus* G1w11	30.4	[28.0–32.9]	1.13
*G. thermodenitrificans* DSM465	26.3	[24.0–28.8]	3.55

## Data Availability

The original contributions presented in the study are included in the article; further inquiries can be directed to the corresponding authors.

## Supplementary Materials

The following supporting information can be downloaded at: https://www.mdpi.com/article/10.3390/microorganisms13061374/s1, Table S1: The type strains of the genera *Bacillus* and *Geobacillus* used to build the phylogenetic tree. Table S2: The complete genomes of type strains from the genera *Bacillus* and *Geobacillus* used to construct the phylogenetic tree. Table S3: A list of genes and markers extracted from complete genomes using the AMPHORA2 tool for phylogenetic tree reconstruction. Table S4: The phenotypic characteristics of *Geobacillus* sp. G4. Table S5: A comparison of the 16S rRNA gene of *Geobacillus* sp. G4 with the NCBI database. Table S6: An analysis of the annotation of possible antibiotic resistance genes. Table S7: An analysis of biosynthetic gene clusters for secondary metabolites. Table S8: Genes associated with the functional categories: heat-shock response, DNA repair and supercoiling, oxidative stress, and Carbon starvation identified in the genomes of *Geobacillus* sp. G4, *G. kaustophilus* HTA426, *G. kaustophilus* NBRC12445, and *G. thermoleovorans* KCTC3570. Figure S1: Phylogenomic tree constructed from type strains of Geobacillus and Bacillus species, retrieved from NCBI (GenBank). Figure S2: Metabolic pathways displaying genes and enzymes of interest annotated in the genome of *G. thermoleovorans* G4.
