# Supplementary material for: Functional Characterization, Genome Assembly, and Annotation of Geobacillus sp. G4 Isolated from a Geothermal Field in Tacna, Peru
Source: Microorganisms. 2025 Jun 13;13(6):1374. doi: 10.3390/microorganisms13061374 (PMC12196064; doi:10.3390/microorganisms13061374)
Supplement: Supplementary file 1 [file microorganisms-13-01374-s001.zip › microorganisms-3611703-supplementary.pdf]

SUPPLEMENTARY MATERIAL

Supplementary Table S1. Type strains of the genera *Bacillus* and *Geobacillus* used to build the phylogenetic tree, with their corresponding GenBank accession numbers. For tree construction, the 16S rRNA gene was extracted from the complete genomes using the barrnap v0.9 tool (https://github.com/tseemann/barrnap).

GenBank accession number	Type strains
JBEPJY010000049.1	***Geobacillus* sp. G4**
CP120681.1	*Bacillus subtilis* DSM10
CP014335.1	*Geobacillus thermoleovorans* KCTC3570
CP038860.1	*Geobacillus kaustophilus* NBRC 102445
BA000043.1	*Geobacillus kaustophilus* HTA 426
JPOI01000001.1	*Geobacillus vulcani* PSS1
MQMG01000129.1	*Geobacillus proteiniphilus* strain 1017
CP128494.1	*Geobacillus stearothermophilus* ATCC 12980
NEWK01000001.1	*Geobacillus thermocatenulatus* BGSC 93A1
BCQG01000022.1	*Geobacillus jurassicus* NBRC 107829
JPYA02000002.1	*Geobacillus icigianus* G1w11
NEWL01000009.1	*Geobacillus uzenensis* BGSC92A1
CP014342.1	*Geobacillus subterraneus* KCTC 3922
AYKT01000045.1	*Geobacillus thermodenitrificans* DSM 465

Supplementary Table S2. Complete genomes of type strains from the genera *Bacillus* and *Geobacillus* used to construct the phylogenetic tree, along with their corresponding GenBank accession numbers.

GenBank accession number	Type strains
GCA_040327975.1	***Geobacillus* sp. G4**
GCA_029537415.1	*Bacillus subtilis* DSM10
GCA_001610955.1	*Geobacillus thermoleovorans* KCTC3570
GCA_005160065.1	*Geobacillus kaustophilus* NBRC 102445
GCA_000009785.1	*Geobacillus kaustophilus* HTA 426
GCA_000733845.1	*Geobacillus vulcani* PSS1
GCA_001908025.1	*Geobacillus proteiniphilus* strain 1017
GCA_030369615.1	*Geobacillus stearothermophilus* ATCC 12980
GCA_002217655.1	*Geobacillus thermocatenulatus* BGSC 93A1
GCA_001544315.1	*Geobacillus jurassicus* NBRC 107829
GCA_000750005.2	*Geobacillus icigianus* G1w11
GCA_002217665.1	*Geobacillus uzenensis* BGSC92A1
GCA_001618685.1	*Geobacillus subterraneus* KCTC 3922
GCA_000496575.1	*Geobacillus thermodenitrificans* DSM 465

Supplementary Table S3. List of genes and markers extracted from complete genomes using the AMPHORA2 tool for phylogenetic tree reconstruction. Available for access: (https://github.com/wu-lab-uva/AMPHORA2/blob/master/Marker/marker.list#L1).

Gene	Organisms	Protein
rplA	Bacteria	50S ribosomal subunit protein L1
rplB	Bacteria	50S ribosomal subunit protein L2
rplC	Bacteria	50S ribosomal subunit protein L3
rplD	Bacteria	50S ribosomal subunit protein L4
rplE	Bacteria	50S ribosomal subunit protein L5
rplF	Bacteria	50S ribosomal subunit protein L6
rplK	Bacteria	50S ribosomal subunit protein L11
rplL	Bacteria	50S ribosomal subunit protein L7/L12
rplM	Bacteria	50S ribosomal subunit protein L13
rplN	Bacteria	50S ribosomal subunit protein L14
rplP	Bacteria	50S ribosomal subunit protein L16
rplS	Bacteria	50S ribosomal subunit protein L19
rplT	Bacteria	50S ribosomal subunit protein L20
rpmA	Bacteria	50S ribosomal subunit protein L27
rpsB	Bacteria	30S ribosomal subunit protein S2
rpsC	Bacteria	30S ribosomal subunit protein S3
rpsE	Bacteria	30S ribosomal subunit protein S5
rpsI	Bacteria	30S ribosomal subunit protein S9
rpsJ	Bacteria	30S ribosomal subunit protein S10
rpsK	Bacteria	30S ribosomal subunit protein S11
rpsM	Bacteria	30S ribosomal subunit protein S13
rpsS	Bacteria	30S ribosomal subunit protein S19
tsf	Bacteria	protein chain elongation factor EF-Ts
frr	Bacteria	ribosome releasing factor
infC	Bacteria	protein chain initiation factor IF-3
smpB	Bacteria	small protein B
rpoB	Bacteria	RNA polymerase beta subunit
nusA	Bacteria	transcription pausing L factor
dnaG	Bacteria	DNA primase
pgk	Bacteria	phosphoglycerate kinase
pyrG	Bacteria	CTP synthetase

Supplementary Table S4. Phenotypic characteristics of *Geobacillus* sp. G4. All phenotypic data were determined in this study.

Physiological and biochemical characterization of *Geobacillus* sp. G4		
Colonies		Shiny, circular and yellowish-white
Average colonies		≈ 1.4 mm
Morphology		Rods
Gram staining		Positive
Endospore formation		+
Motility		-
Oxygen requirement		aerobic
Growth	Temperature (°C)	50 - 70
	pH	6.0 – 7.5
	NaCl (%)	0 – 2.5
Hydrolysis of	CMC	-
	Starch	+
	Pectin	+
	Cellulose	-
	Urea	-
	Casein	+
	Lactose	+
	Xylose	+
	Glucose	+
	Sucrose	+
	Cellobiose	-
	Maltose	+
	Glycerol	+

Supplementary Table S5. Comparison of the 16S rRNA gene of *Geobacillus* sp. G4 with the NCBI (core_nt) database using the blastn tool.

Scientific Name	Max Score	Total Score	Query Cover	Per. Ident	Accession number
Geobacillus kaustophilus BGSC 90A1	2863	2863	100%	99.87%	NR_115285.2
Geobacillus thermoleovorans LEH-1	2787	2787	97%	99.80%	NR_036985.1
Geobacillus thermoleovorans BGSC 96A1	2846	2846	100%	99.61%	NR_115286.2
Bacillus caldotenax DSM 406	2784	2784	97%	99.61%	NR_126321.1
Geobacillus kaustophilus NBRC 102445	2715	2715	95%	99.60%	NR_114089.1
Geobacillus vulcani 3S-1	2785	2785	98%	99.48%	NR_025426.1
Bacillus caldolyticus DSM 405	2763	2763	97%	99.41%	NR_126322.1
Geobacillus lituanicus N-3	2747	2747	97%	99.21%	NR_025657.1
Geobacillus kaustophilus BD 53	2575	2575	92%	99.09%	NR_029224.1
Bacillus caldovelox DSM 411	2743	2743	97%	99.08%	NR_126323.1
Geobacillus zalihae NBRC 101842	2678	2678	95%	99.06%	NR_114014.1

Supplementary Table S6. Analysis of the annotation of possible antibiotic resistance genes using the Antibiotic Resistance Genes Database (ARDB) within the RGI v6.0.3 portal on the CARD v3.2.8 platform. *Geobacillus* sp. G4; G.K HTA425 – *G. kaustophilus* HTA426; G.K NBRC102445 – *G. kaustophilus* NBRC102445; and G.T KCTC3570 – *G. thermoleovorans* KCTC3570. Similarity in percentage (%).

**Gene**	**Family**	**Drug Class**	**Resistance Mechanism**	***Geobacillus* sp. G4**	**G. K HTA425 (%)**	**G. K NBRC102445 (%)**	**G. T KCTC3570 (%)**
vanY_in_vanB_cl	vanY, glycopeptide resistance gene cluster	Glycopeptide antibiotic	Antibiotic target alteration	36.41	34.15	34.15	34.15
vanT_in_vanG_cl	Glycopeptide resistance gene cluster, vanT	Glycopeptide antibiotic	Antibiotic target alteration	34.15	35.9	35.9	35.9

Supplementary Table S7. Analysis of biosynthetic gene clusters for secondary metabolites performed by the AntiSMASH server (v. 7.0.1). *Geobacillus* sp. G4; G.K HTA425 – *G. kaustophilus* HTA425; G.K NBRC102445 – *G. kaustophilus* NBRC102445; and G.T KCTC3570 – *G. thermoleovorans* KCTC3570.

**Secondary Metabolites**				
	**Clusters number**			
**Type**	***Geobacillus* sp. G4**	**G.K HTA 425**	**G.K NBRC 102445**	**G.T KCTC 3570**
Betalactone	1	1	1	1
T3PKS	1	1	1	1
Lanthipeptide-class-i	1	1	1	-
NI-siderophore	1	-	-	-
Terpene	1	-	-	1
Fengycin	1	-	-	-
RiPP-like	1	-	-	-

Supplementary Table S8. Genes associated with the functional categories "Heat-shock response," "DNA repair and supercoiling," "Oxidative stress," and "Carbon starvation" identified in the genomes of *Geobacillus* sp. G4, *G. kaustophilus* HTA426, *G. kaustophilus* NBRC12445, and *G. thermoleovorans* KCTC3570. The presence or absence of these genes is shown in Figure 9. The table includes the gene name, encoded product, COG number (for functional reference), species, and functional category.

**Gene**	**Product**	**COG***	**Species**	**Category**
cspB	Cold shock protein CspB	COG1278	*Geobacillus* sp. G4	Heat-shock response
hrcA	Heat-inducible transcription repressor HrcA	COG1420	*Geobacillus* sp. G4	Heat-shock response
grpE	Protein GrpE	COG0576	*Geobacillus* sp. G4	Heat-shock response
dnaK	Chaperone protein DnaK	COG0443	*Geobacillus* sp. G4	Heat-shock response
dnaJ	Chaperone protein DnaJ	COG0484	*Geobacillus* sp. G4	Heat-shock response
hspA	Spore protein SP21	COG0071	*Geobacillus* sp. G4	Heat-shock response
clpB	Chaperone protein ClpB	COG0542	*Geobacillus* sp. G4	Heat-shock response
ibpB	Small heat shock protein IbpB	COG0071	*Geobacillus* sp. G4	Heat-shock response
clpC	Negative regulator of genetic competence ClpC/MecB	COG0542	*Geobacillus* sp. G4	Heat-shock response

clpX	ATP-dependent Clp protease ATP-binding subunit ClpX	COG1219	*Geobacillus* sp. G4	Heat-shock response
clpE	ATP-dependent Clp protease ATP-binding subunit ClpE	COG0542	*Geobacillus* sp. G4	Heat-shock response
clpP	ATP-dependent Clp protease proteolytic subunit	COG0740	*Geobacillus* sp. G4	Heat-shock response
ctsr	Transcriptional regulator CtsR	COG1846	*Geobacillus* sp. G4	Heat-shock response
ftsH	ATP-dependent zinc metalloprotease FtsH	COG0465	*Geobacillus* sp. G4	Heat-shock response
mutL	DNA mismatch repair protein MutL	COG0323	*Geobacillus* sp. G4	DNA repair and supercoiling
mutS	DNA mismatch repair protein MutS	COG0249	*Geobacillus* sp. G4	DNA repair and supercoiling
mutY	Adenine DNA glycosylase	COG1194	*Geobacillus* sp. G4	DNA repair and supercoiling
mutM	Formamidopyrimidine-DNA glycosylase	COG0266	*Geobacillus* sp. G4	DNA repair and supercoiling
mutX	8-oxo-dGTP diphosphatase	COG0494	*Geobacillus* sp. G4	DNA repair and supercoiling
recA_1	Protein RecA	COG0468	*Geobacillus* sp. G4	DNA repair and supercoiling
recA_2	Protein RecA	COG0468	*Geobacillus* sp. G4	DNA repair and supercoiling
recR	Recombination protein RecR	COG0353	*Geobacillus* sp. G4	DNA repair and supercoiling
recO	DNA repair protein RecO	COG1381	*Geobacillus* sp. G4	DNA repair and supercoiling
topA	DNA topoisomerase 1	COG0550	*Geobacillus* sp. G4	DNA repair and supercoiling

ptsI	Phosphoenolpyruvate-protein phosphotransferase	COG1080	*Geobacillus* sp. G4	DNA repair and supercoiling
murB	UDP-N-acetylenolpyruvoylglucosamine reductase	COG0812	*Geobacillus* sp. G4	DNA repair and supercoiling
ruvB	Holliday junction ATP-dependent DNA helicase RuvB	COG2255	*Geobacillus* sp. G4	DNA repair and supercoiling
ruvA	Holliday junction ATP-dependent DNA helicase RuvA	COG0632	*Geobacillus* sp. G4	DNA repair and supercoiling
uvrC	UvrABC system protein C	COG0322	*Geobacillus* sp. G4	DNA repair and supercoiling
menD	2-succinyl-5-enolpyruvyl-6-hydroxy-3-cyclohexene-1-carboxylate synthase	COG1165	*Geobacillus* sp. G4	DNA repair and supercoiling
yqfL	Putative pyruvate, phosphate dikinase regulatory protein	COG1806	*Geobacillus* sp. G4	DNA repair and supercoiling
recN	DNA repair protein RecN	COG0497	*Geobacillus* sp. G4	DNA repair and supercoiling
pdhC_3	Dihydrolipoyllysine-residue acetyltransferase component of pyruvate dehydrogenase complex	COG0508	*Geobacillus* sp. G4	DNA repair and supercoiling
ghrB	Glyoxylate/hydroxypyruvate reductase B	COG1052	*Geobacillus* sp. G4	DNA repair and supercoiling
uvrA	UvrABC system protein A	COG0178	*Geobacillus* sp. G4	DNA repair and supercoiling
uvrB_1	UvrABC system protein B	COG0556	*Geobacillus* sp. G4	DNA repair and supercoiling
hyi	Hydroxypyruvate isomerase	COG3622	*Geobacillus* sp. G4	DNA repair and supercoiling

sseA	3-mercaptopyruvate sulfurtransferase	COG2897	*Geobacillus* sp. G4	DNA repair and supercoiling
topB_1	DNA topoisomerase 3	COG0550	*Geobacillus* sp. G4	DNA repair and supercoiling
uvrB_2	UvrABC system protein B	COG0556	*Geobacillus* sp. G4	DNA repair and supercoiling
gyrB	DNA gyrase subunit B	COG0187	*Geobacillus* sp. G4	DNA repair and supercoiling
gyrA	DNA gyrase subunit A	COG0188	*Geobacillus* sp. G4	DNA repair and supercoiling
topB_2	DNA topoisomerase 3	COG0550	*Geobacillus* sp. G4	DNA repair and supercoiling
hpr_2	Hydroxypyruvate reductase	COG1052	*Geobacillus* sp. G4	DNA repair and supercoiling
pdhC_4	Dihydrolipoyllysine-residue acetyltransferase component of pyruvate dehydrogenase complex	COG0508	*Geobacillus* sp. G4	DNA repair and supercoiling
radA	DNA repair protein RadA	COG1066	*Geobacillus* sp. G4	DNA repair and supercoiling
tpa_1	Taurine--pyruvate aminotransferase	COG0161	*Geobacillus* sp. G4	DNA repair and supercoiling
tpa_2	Taurine--pyruvate aminotransferase	COG0161	*Geobacillus* sp. G4	DNA repair and supercoiling
parE	DNA topoisomerase 4 subunit B	COG0187	*Geobacillus* sp. G4	DNA repair and supercoiling
uvrB_3	UvrABC system protein B	COG0556	*Geobacillus* sp. G4	DNA repair and supercoiling

ahpF	NADH dehydrogenase	COG3634	*Geobacillus* sp. G4	Oxidative stress
ahpC	Alkyl hydroperoxide reductase C	COG0450	*Geobacillus* sp. G4	Oxidative stress
trxA	Thioredoxin	COG0526	*Geobacillus* sp. G4	Oxidative stress
bcp	Putative peroxiredoxin bcp	COG1225	*Geobacillus* sp. G4	Oxidative stress
dps	General stress protein 20U	COG0783	*Geobacillus* sp. G4	Oxidative stress
ctc	General stress protein CTC	COG1825	*Geobacillus* sp. G4	Oxidative stress
hslO	Redox-regulated molecular chaperone, HSP33 family	COG1281	*Geobacillus* sp. G4	Oxidative stress
msrA	Peptide methionine sulfoxide reductase MsrA	COG0225	*Geobacillus* sp. G4	Oxidative stress
tpx	Thiol peroxidase	COG2077	*Geobacillus* sp. G4	Oxidative stress
sodA	Superoxide dismutase [Mn]	COG0605	*Geobacillus* sp. G4	Oxidative stress
ydbP	Thioredoxin-like protein YdbP	COG0526	*Geobacillus* sp. G4	Oxidative stress
perR	Peroxide operon regulator	COG0735	*Geobacillus* sp. G4	Oxidative stress
yojM	Superoxide dismutase-like protein YojM	COG2032	*Geobacillus* sp. G4	Oxidative stress
trxB	Thioredoxin reductase	COG0492	*Geobacillus* sp. G4	Oxidative stress
katG	Catalase-peroxidase	COG0376	*Geobacillus* sp. G4	Oxidative stress
cstA	Peptide transporter CstA	COG1966	*Geobacillus* sp. G4	Carbon starvation
relA	GTP pyrophosphokinase	COG0317	*Geobacillus* sp. G4	Carbon starvation
csrA	sRNA-binding carbon storage regulator CsrA	COG1551	*Geobacillus* sp. G4	Carbon starvation
ackA	Acetate kinase	COG0282	*Geobacillus* sp. G4	Carbon starvation
aceA	Isocitrate lyase	COG2224	*Geobacillus* sp. G4	Carbon starvation

radA	DNA repair protein RadA	COG1066	*Geobacillus thermoleovorans* KCTC3570	DNA repair and supercoiling
pdhR	Pyruvate dehydrogenase complex repressor	COG2186	*Geobacillus thermoleovorans* KCTC3570	DNA repair and supercoiling
murB	UDP-N-acetylenolpyruvoylglucosamine reductase	COG0812	*Geobacillus thermoleovorans* KCTC3570	DNA repair and supercoiling
ptsI	Phosphoenolpyruvate-protein phosphotransferase	COG1080	*Geobacillus thermoleovorans* KCTC3570	DNA repair and supercoiling
topA	DNA topoisomerase 1	COG0550	*Geobacillus thermoleovorans* KCTC3570	DNA repair and supercoiling
recA_1	Protein RecA	COG0468	*Geobacillus thermoleovorans* KCTC3570	DNA repair and supercoiling
recA_2	Protein RecA	COG0468	*Geobacillus thermoleovorans* KCTC3570	DNA repair and supercoiling
mutS	DNA mismatch repair protein MutS	COG0249	*Geobacillus thermoleovorans* KCTC3570	DNA repair and supercoiling
mutL	DNA mismatch repair protein MutL	COG0323	*Geobacillus thermoleovorans* KCTC3570	DNA repair and supercoiling
tpa	Taurine--pyruvate aminotransferase	COG0161	*Geobacillus thermoleovorans* KCTC3570	DNA repair and supercoiling
hyi	Hydroxypyruvate isomerase	COG3622	*Geobacillus thermoleovorans* KCTC3570	DNA repair and supercoiling
sseA	3-mercaptopyruvate sulfurtransferase	COG2897	*Geobacillus thermoleovorans* KCTC3570	DNA repair and supercoiling
topB_1	DNA topoisomerase 3	COG0550	*Geobacillus thermoleovorans* KCTC3570	DNA repair and supercoiling

parE	DNA topoisomerase 4 subunit B	COG0187	*Geobacillus thermoleovorans* KCTC3570	DNA repair and supercoiling
topB_2	DNA topoisomerase 3	COG0550	*Geobacillus thermoleovorans* KCTC3570	DNA repair and supercoiling
recN	DNA repair protein RecN	COG0497	*Geobacillus thermoleovorans* KCTC3570	DNA repair and supercoiling
yqfL	Putative pyruvate, phosphate dikinase regulatory protein	COG1806	*Geobacillus thermoleovorans* KCTC3570	DNA repair and supercoiling
ruvB	Holliday junction ATP-dependent DNA helicase RuvB	COG2255	*Geobacillus thermoleovorans* KCTC3570	DNA repair and supercoiling
ruvA	Holliday junction ATP-dependent DNA helicase RuvA	COG0632	*Geobacillus thermoleovorans* KCTC3570	DNA repair and supercoiling
uvrC	UvrABC system protein C	COG0322	*Geobacillus thermoleovorans* KCTC3570	DNA repair and supercoiling
menD	2-succinyl-5-enolpyruvyl-6-hydroxy-3-cyclohexene-1-carboxylate synthase	COG1165	*Geobacillus thermoleovorans* KCTC3570	DNA repair and supercoiling
ghrB	Glyoxylate/hydroxypyruvate reductase B	COG1052	*Geobacillus thermoleovorans* KCTC3570	DNA repair and supercoiling
uvrA	UvrABC system protein A	COG0178	*Geobacillus thermoleovorans* KCTC3570	DNA repair and supercoiling
uvrB	UvrABC system protein B	COG0556	*Geobacillus thermoleovorans* KCTC3570	DNA repair and supercoiling
pdhC_4	Dihydrolipoyllysine-residue acetyltransferase component of pyruvate dehydrogenase complex	COG0508	*Geobacillus thermoleovorans* KCTC3570	DNA repair and supercoiling
gyrB	DNA gyrase subunit B	COG0187	*Geobacillus thermoleovorans* KCTC3570	DNA repair and supercoiling

gyrA	DNA gyrase subunit A	COG0188	*Geobacillus thermoleovorans* KCTC3570	DNA repair and supercoiling
ibpB	Small heat shock protein IbpB	COG0071	*Geobacillus thermoleovorans* KCTC3570	Heat-shock response
clpB	Chaperone protein ClpB	COG0542	*Geobacillus thermoleovorans* KCTC3570	Heat-shock response
cspB	Cold shock protein CspB	COG1278	*Geobacillus thermoleovorans* KCTC3570	Heat-shock response
hspA	Spore protein SP21	COG0071	*Geobacillus thermoleovorans* KCTC3570	Heat-shock response
dnaK	Chaperone protein DnaK	COG0443	*Geobacillus thermoleovorans* KCTC3570	Heat-shock response
dnaJ	Chaperone protein DnaJ	COG0484	*Geobacillus thermoleovorans* KCTC3570	Heat-shock response
grpE	Protein GrpE	COG0576	*Geobacillus thermoleovorans* KCTC3570	Heat-shock response
hrcA	Heat-inducible transcription repressor HrcA	COG1420	*Geobacillus thermoleovorans* KCTC3570	Heat-shock response
clpC	Negative regulator of genetic competence ClpC/MecB	COG0542	*Geobacillus thermoleovorans* KCTC3570	Heat-shock response
ydbP	Thioredoxin-like protein YdbP	COG0526	*Geobacillus thermoleovorans* KCTC3570	Oxidative stress
katG	Catalase-peroxidase	COG0376	*Geobacillus thermoleovorans* KCTC3570	Oxidative stress
sodA	Superoxide dismutase [Mn]	COG0605	*Geobacillus thermoleovorans* KCTC3570	Oxidative stress

ahpF	NADH dehydrogenase	COG3634	*Geobacillus thermoleovorans* KCTC3570	Oxidative stress
ahpC	Alkyl hydroperoxide reductase C	COG0450	*Geobacillus thermoleovorans* KCTC3570	Oxidative stress
trxA	Thioredoxin	COG0526	*Geobacillus thermoleovorans* KCTC3570	Oxidative stress
tpx	Thiol peroxidase	COG2077	*Geobacillus thermoleovorans* KCTC3570	Oxidative stress
yojM	Superoxide dismutase-like protein YojM	COG2032	*Geobacillus thermoleovorans* KCTC3570	Oxidative stress
trxB	Thioredoxin reductase	COG0492	*Geobacillus thermoleovorans* KCTC3570	Oxidative stress
cstA	Peptide transporter CstA	COG1966	*Geobacillus thermoleovorans* KCTC3570	Carbon starvation
gyrB	DNA gyrase subunit B	COG0187	*Geobacillus kaustophilus* HTA426	DNA repair and supercoiling
gyrA	DNA gyrase subunit A	COG0188	*Geobacillus kaustophilus* HTA426	DNA repair and supercoiling
radA	DNA repair protein RadA	COG1066	*Geobacillus kaustophilus* HTA426	DNA repair and supercoiling
uvrB_1	UvrABC system protein B	COG0556	*Geobacillus kaustophilus* HTA426	DNA repair and supercoiling
murB	UDP-N-acetylenolpyruvoylglucosamine reductase	COG0812	*Geobacillus kaustophilus* HTA426	DNA repair and supercoiling
ptsI	Phosphoenolpyruvate-protein phosphotransferase	COG1080	*Geobacillus kaustophilus* HTA426	DNA repair and supercoiling

topA	DNA topoisomerase 1	COG0550	*Geobacillus kaustophilus* HTA426	DNA repair and supercoiling
recA_1	Protein RecA	COG0468	*Geobacillus kaustophilus* HTA426	DNA repair and supercoiling
recA_2	Protein RecA	COG0468	*Geobacillus kaustophilus* HTA426	DNA repair and supercoiling
recN	DNA repair protein RecN	COG0497	*Geobacillus kaustophilus* HTA426	DNA repair and supercoiling
recR	Recombination protein RecR	COG0353	*Geobacillus kaustophilus* HTA426	DNA repair and supercoiling
recO	DNA repair protein RecO	COG1381	*Geobacillus kaustophilus* HTA426	DNA repair and supercoiling
mutS	DNA mismatch repair protein MutS	COG0249	*Geobacillus kaustophilus* HTA426	DNA repair and supercoiling
mutL	DNA mismatch repair protein MutL	COG0323	*Geobacillus kaustophilus* HTA426	DNA repair and supercoiling
mutY	Adenine DNA glycosylase	COG1194	*Geobacillus kaustophilus* HTA426	DNA repair and supercoiling
mutM	Formamidopyrimidine-DNA glycosylase	COG0266	*Geobacillus kaustophilus* HTA426	DNA repair and supercoiling
mutX	8-oxo-dGTP diphosphatase	COG0494	*Geobacillus kaustophilus* HTA426	DNA repair and supercoiling
tpa	Taurine--pyruvate aminotransferase	COG0161	*Geobacillus kaustophilus* HTA426	DNA repair and supercoiling
hyi	Hydroxypyruvate isomerase	COG3622	*Geobacillus kaustophilus* HTA426	DNA repair and supercoiling

sseA	3-mercaptopyruvate sulfurtransferase	COG2897	*Geobacillus kaustophilus* HTA426	DNA repair and supercoiling
topB_1	DNA topoisomerase 3	COG0550	*Geobacillus kaustophilus* HTA426	DNA repair and supercoiling
parE	DNA topoisomerase 4 subunit B	COG0187	*Geobacillus kaustophilus* HTA426	DNA repair and supercoiling
topB_2	DNA topoisomerase 3	COG0550	*Geobacillus kaustophilus* HTA426	DNA repair and supercoiling
hpr_2	Hydroxypyruvate reductase	COG1052	*Geobacillus kaustophilus* HTA426	DNA repair and supercoiling
pdhB_3	Pyruvate dehydrogenase E1 component subunit beta	COG0022	*Geobacillus kaustophilus* HTA426	DNA repair and supercoiling
uvrB_2	UvrABC system protein B	COG0556	*Geobacillus kaustophilus* HTA426	DNA repair and supercoiling
recN	DNA repair protein RecN	COG0497	*Geobacillus kaustophilus* HTA426	DNA repair and supercoiling
yqfL	Putative pyruvate, phosphate dikinase regulatory protein	COG1806	*Geobacillus kaustophilus* HTA426	DNA repair and supercoiling
ruvB	Holliday junction ATP-dependent DNA helicase RuvB	COG2255	*Geobacillus kaustophilus* HTA426	DNA repair and supercoiling
ruvA	Holliday junction ATP-dependent DNA helicase RuvA	COG0632	*Geobacillus kaustophilus* HTA426	DNA repair and supercoiling
uvrC	UvrABC system protein C	COG0322	*Geobacillus kaustophilus* HTA426	DNA repair and supercoiling
menD	2-succinyl-5-enolpyruvyl-6-hydroxy-3-cyclohexene-1-carboxylate synthase	COG1165	*Geobacillus kaustophilus* HTA426	DNA repair and supercoiling

ghrB	Glyoxylate/hydroxypyruvate reductase B	COG1052	*Geobacillus kaustophilus* HTA426	DNA repair and supercoiling
uvrA	UvrABC system protein A	COG0178	*Geobacillus kaustophilus* HTA426	DNA repair and supercoiling
uvrB_3	UvrABC system protein B	COG0556	*Geobacillus kaustophilus* HTA426	DNA repair and supercoiling
pdhC_4	Dihydrolipoyllysine-residue acetyltransferase component of pyruvate dehydrogenase complex	COG0508	*Geobacillus kaustophilus* HTA426	DNA repair and supercoiling
ibpB	Small heat shock protein IbpB	COG0071	*Geobacillus kaustophilus* HTA426	Heat-shock response
clpB	Chaperone protein ClpB	COG0542	*Geobacillus kaustophilus* HTA426	Heat-shock response
cspB	Cold shock protein CspB	COG1278	*Geobacillus kaustophilus* HTA426	Heat-shock response
hspA	Spore protein SP21	COG0071	*Geobacillus kaustophilus* HTA426	Heat-shock response
dnaK	Chaperone protein DnaK	COG0443	*Geobacillus kaustophilus* HTA426	Heat-shock response
dnaJ	Chaperone protein DnaJ	COG0484	*Geobacillus kaustophilus* HTA426	Heat-shock response
grpE	Protein GrpE	COG0576	*Geobacillus kaustophilus* HTA426	Heat-shock response
hrcA	Heat-inducible transcription repressor HrcA	COG1420	*Geobacillus kaustophilus* HTA426	Heat-shock response
clpC	Negative regulator of genetic competence ClpC/MecB	COG0542	*Geobacillus kaustophilus* HTA426	Heat-shock response
clpE	ATP-dependent Clp protease ATP-binding subunit ClpE	COG0542	*Geobacillus kaustophilus* HTA426	Heat-shock response
clpP	ATP-dependent Clp protease proteolytic subunit	COG0740	*Geobacillus kaustophilus* HTA426	Heat-shock response
ydbP	Thioredoxin-like protein YdbP	COG0526	*Geobacillus kaustophilus* HTA426	Oxidative stress
katG	Catalase-peroxidase	COG0376	*Geobacillus kaustophilus* HTA426	Oxidative stress
ohrA	Organic hydroperoxide resistance protein OhrA	COG1764	*Geobacillus kaustophilus* HTA426	Oxidative stress

sodA	Superoxide dismutase [Mn]	COG0605	*Geobacillus kaustophilus* HTA426	Oxidative stress
bcp	Putative peroxiredoxin bcp	COG1225	*Geobacillus kaustophilus* HTA426	Oxidative stress
ctc	General stress protein CTC	COG1825	*Geobacillus kaustophilus* HTA426	Oxidative stress
msrA	Peptide methionine sulfoxide reductase MsrA	COG0225	*Geobacillus kaustophilus* HTA426	Oxidative stress
ahpF	NADH dehydrogenase	COG3634	*Geobacillus kaustophilus* HTA426	Oxidative stress
ahpC	Alkyl hydroperoxide reductase C	COG0450	*Geobacillus kaustophilus* HTA426	Oxidative stress
trxA	Thioredoxin	COG0526	*Geobacillus kaustophilus* HTA426	Oxidative stress
dps	General stress protein 20U	COG0783	*Geobacillus kaustophilus* HTA426	Oxidative stress
perR	Peroxide operon regulator	COG0735	*Geobacillus kaustophilus* HTA426	Oxidative stress
tpx	Thiol peroxidase	COG2077	*Geobacillus kaustophilus* HTA426	Oxidative stress
yojM	Superoxide dismutase-like protein YojM	COG2032	*Geobacillus kaustophilus* HTA426	Oxidative stress
trxB	Thioredoxin reductase	COG0492	*Geobacillus kaustophilus* HTA426	Oxidative stress
hslO	Redox-regulated molecular chaperone, HSP33 family	COG1281	*Geobacillus kaustophilus* HTA426	Oxidative stress
relA	GTP pyrophosphokinase	COG0317	*Geobacillus kaustophilus* HTA426	Carbon starvation
cstA	Peptide transporter CstA	COG1966	*Geobacillus kaustophilus* HTA426	Carbon starvation
csrA	sRNA-binding carbon storage regulator CsrA	COG1551	*Geobacillus kaustophilus* HTA426	Carbon starvation
ackA	Acetate kinase	COG0282	*Geobacillus kaustophilus* HTA426	Carbon starvation
aceA	Isocitrate lyase	COG2224	*Geobacillus kaustophilus* HTA426	Carbon starvation
ghrB	Glyoxylate/hydroxypyruvate reductase B	COG1052	*Geobacillus kaustophilus* NBRC12445	DNA repair and supercoiling

uvrA	UvrABC system protein A	COG0178	*Geobacillus kaustophilus* NBRC12445	DNA repair and supercoiling
uvrB_1	UvrABC system protein B	COG0556	*Geobacillus kaustophilus* NBRC12445	DNA repair and supercoiling
pdhC_1	Dihydrolipoyllysine-residue acetyltransferase component of pyruvate dehydrogenase complex	COG0508	*Geobacillus kaustophilus* NBRC12445	DNA repair and supercoiling
gyrB	DNA gyrase subunit B	COG0187	*Geobacillus kaustophilus* NBRC12445	DNA repair and supercoiling
gyrA	DNA gyrase subunit A	COG0188	*Geobacillus kaustophilus* NBRC12445	DNA repair and supercoiling
radA	DNA repair protein RadA	COG1066	*Geobacillus kaustophilus* NBRC12445	DNA repair and supercoiling
uvrB_2	UvrABC system protein B	COG0556	*Geobacillus kaustophilus* NBRC12445	DNA repair and supercoiling
pdhC_2	Dihydrolipoyllysine-residue acetyltransferase component of pyruvate dehydrogenase complex	COG0508	*Geobacillus kaustophilus* NBRC12445	DNA repair and supercoiling
murB	UDP-N-acetylenolpyruvoylglucosamine reductase	COG0812	*Geobacillus kaustophilus* NBRC12445	DNA repair and supercoiling
ptsI	Phosphoenolpyruvate-protein phosphotransferase	COG1080	*Geobacillus kaustophilus* NBRC12445	DNA repair and supercoiling
topA_1	DNA topoisomerase 1	COG0550	*Geobacillus kaustophilus* NBRC12445	DNA repair and supercoiling
recA_1	Protein RecA	COG0468	*Geobacillus kaustophilus* NBRC12445	DNA repair and supercoiling
recA_2	Protein RecA	COG0468	*Geobacillus kaustophilus* NBRC12445	DNA repair and supercoiling

mutS	DNA mismatch repair protein MutS	COG0249	*Geobacillus kaustophilus* NBRC12445	DNA repair and supercoiling
mutL_1	DNA mismatch repair protein MutL	COG0323	*Geobacillus kaustophilus* NBRC12445	DNA repair and supercoiling
mutL_2	DNA mismatch repair protein MutL	COG0323	*Geobacillus kaustophilus* NBRC12445	DNA repair and supercoiling
topA_2	DNA topoisomerase 1	COG0550	*Geobacillus kaustophilus* NBRC12445	DNA repair and supercoiling
tpa	Taurine--pyruvate aminotransferase	COG0161	*Geobacillus kaustophilus* NBRC12445	DNA repair and supercoiling
hyi	Hydroxypyruvate isomerase	COG3622	*Geobacillus kaustophilus* NBRC12445	DNA repair and supercoiling
topB_1	DNA topoisomerase 3	COG0550	*Geobacillus kaustophilus* NBRC12445	DNA repair and supercoiling
parC_2	DNA topoisomerase 4 subunit A	COG0188	*Geobacillus kaustophilus* NBRC12445	DNA repair and supercoiling
parE	DNA topoisomerase 4 subunit B	COG0187	*Geobacillus kaustophilus* NBRC12445	DNA repair and supercoiling
topB_2	DNA topoisomerase 3	COG0550	*Geobacillus kaustophilus* NBRC12445	DNA repair and supercoiling
uvrB_3	UvrABC system protein B	COG0556	*Geobacillus kaustophilus* NBRC12445	DNA repair and supercoiling
recN	DNA repair protein RecN	COG0497	*Geobacillus kaustophilus* NBRC12445	DNA repair and supercoiling
yqfL	Putative pyruvate, phosphate dikinase regulatory protein	COG1806	*Geobacillus kaustophilus* NBRC12445	DNA repair and supercoiling

ruvB	Holliday junction ATP-dependent DNA helicase RuvB	COG2255	*Geobacillus kaustophilus* NBRC12445	DNA repair and supercoiling
ruvA	Holliday junction ATP-dependent DNA helicase RuvA	COG0632	*Geobacillus kaustophilus* NBRC12445	DNA repair and supercoiling
uvrC	UvrABC system protein C	COG0322	*Geobacillus kaustophilus* NBRC12445	DNA repair and supercoiling
menD	2-succinyl-5-enolpyruvyl-6-hydroxy-3-cyclohexene-1-carboxylate synthase	COG1165	*Geobacillus kaustophilus* NBRC12445	DNA repair and supercoiling
trxB	Thioredoxin reductase	COG0492	*Geobacillus kaustophilus* NBRC12445	Oxidative stress
ydbP	Thioredoxin-like protein YdbP	COG0526	*Geobacillus kaustophilus* NBRC12445	Oxidative stress
katG	Catalase-peroxidase	COG0376	*Geobacillus kaustophilus* NBRC12445	Oxidative stress
sodA	Superoxide dismutase [Mn]	COG0605	*Geobacillus kaustophilus* NBRC12445	Oxidative stress
ahpF	NADH dehydrogenase	COG3634	*Geobacillus kaustophilus* NBRC12445	Oxidative stress
ahpC	Alkyl hydroperoxide reductase C	COG0450	*Geobacillus kaustophilus* NBRC12445	Oxidative stress
trxA	Thioredoxin	COG0526	*Geobacillus kaustophilus* NBRC12445	Oxidative stress
tpx	Thiol peroxidase	COG2077	*Geobacillus kaustophilus* NBRC12445	Oxidative stress
yojM	Superoxide dismutase-like protein YojM	COG2032	*Geobacillus kaustophilus* NBRC12445	Oxidative stress

ibpB	Small heat shock protein IbpB	COG0071	*Geobacillus kaustophilus* NBRC12445	Heat-shock response
clpB	Chaperone protein ClpB	COG0542	*Geobacillus kaustophilus* NBRC12445	Heat-shock response
cspB	Cold shock protein CspB	COG1278	*Geobacillus kaustophilus* NBRC12445	Heat-shock response
hspA	Spore protein SP21	COG0071	*Geobacillus kaustophilus* NBRC12445	Heat-shock response
dnaK	Chaperone protein DnaK	COG0443	*Geobacillus kaustophilus* NBRC12445	Heat-shock response
grpE	Protein GrpE	COG0576	*Geobacillus kaustophilus* NBRC12445	Heat-shock response
dnaJ	Chaperone protein DnaJ	COG0484	*Geobacillus kaustophilus* NBRC12445	Heat-shock response
hrcA	Heat-inducible transcription repressor HrcA	COG1420	*Geobacillus kaustophilus* NBRC12445	Heat-shock response
clpC	Negative regulator of genetic competence ClpC/MecB	COG0542	*Geobacillus kaustophilus* NBRC12445	Heat-shock response
cstA	Peptide transporter CstA	COG1966	*Geobacillus kaustophilus* NBRC12445	Carbon starvation

*COG: Clusters of Orthologous Groups.

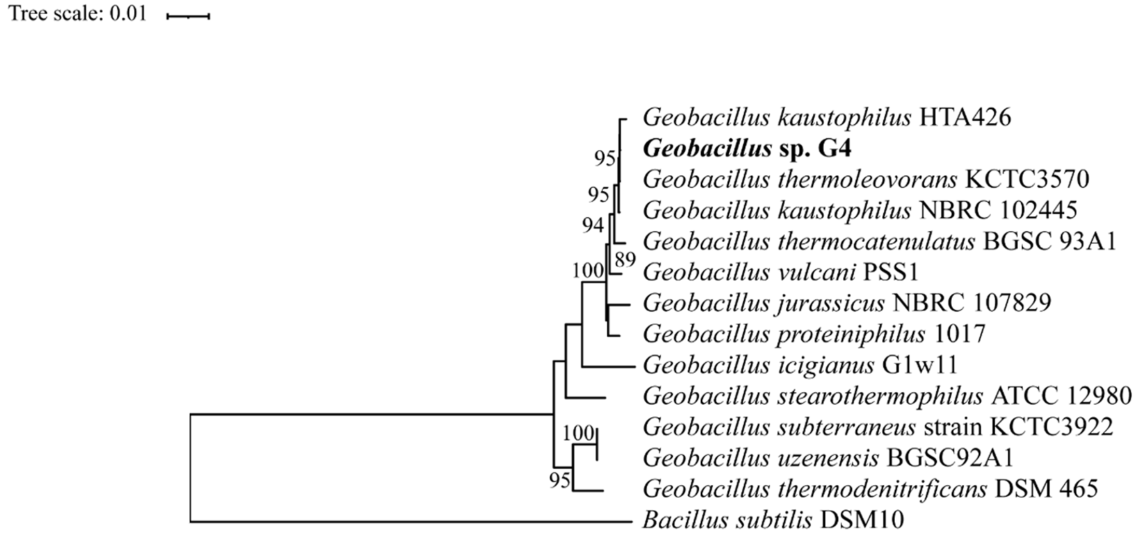

Supplementary Figure S1. Phylogenomic tree constructed from type strains of *Geobacillus* and *Bacillus* species, retrieved from NCBI (GenBank). The accession numbers are available in Supplementary Table S2.

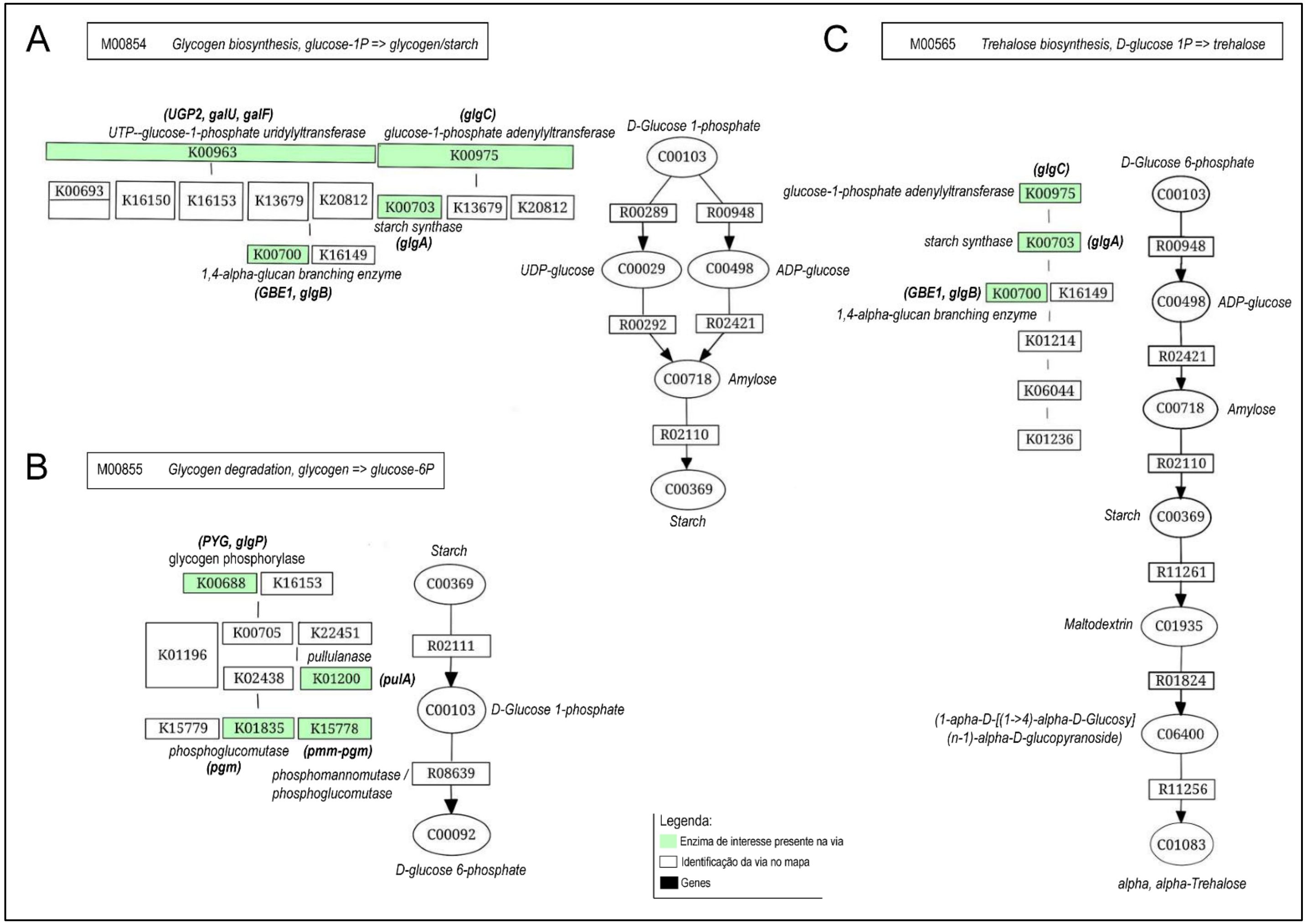

Supplementary Figure S2. Metabolic pathways displaying genes and enzymes of interest annotated in the genome of *G. thermoleovorans* G4. (A) Glycogen biosynthesis (M00854); (B) Glycogen degradation (M00855); (C) Trehalose biosynthesis (M00565). Annotations were performed using the KEGG Mapper tool (www.genome.jp/kegg/mapper), identifying key enzymes involved in the metabolism of sugars and complex carbohydrates.
